# Click chemistry of phenyl 1,2,3-triazole–2-pyridylpiperazine hybrids: synthesis, targeted anticancer activity, molecular modeling and computational studies

**DOI:** 10.1039/d6ra01413e

**Published:** 2026-05-14

**Authors:** Tamer El Malah, Ahmed A. El-Rashedy

**Affiliations:** a Photochemistry Department, Chemical Industries Research Institute, National Research Centre 33 El Buhouth Street, P.O. Box 12622 Cairo Egypt tmara_nrc3000@yahoo.com; b Chemistry of Natural and Microbial Products Department, National Research Centre Dokki 12622 Cairo Egypt; c Department of Organic and Medicinal Chemistry, Faculty of Pharmacy, University of Sadat City Monofia 32897 Egypt

## Abstract

A novel series of phenyl 1,2,3-triazole–2-pyridylpiperazine derivatives (13–23) was synthesized *via* Cu(i)-catalyzed azide–alkyne cycloaddition (CuAAC) click chemistry. The structures of the obtained compounds were confirmed using standard spectroscopic techniques. Their antiproliferative activities were evaluated against human colorectal (HCT-116), liver (HepG-2), and breast (MCF-7) cancer cell lines, along with normal human lung fibroblasts (WI-38) using the MTT assay. Several derivatives exhibited significant cytotoxic activity, with compounds 15 and 18 showing potent inhibitory effects comparable to doxorubicin. Molecular docking and molecular dynamics simulations suggested stable binding interactions of the most active compound with the EGFR receptor. *In silico* drug-likeness and ADME analyses indicated acceptable pharmacokinetic properties for most compounds. These findings suggest that phenyl 1,2,3-triazole–2-pyridylpiperazine derivatives, particularly compound 15, may serve as promising lead candidates for the development of new anticancer agents.

## Abstract 2

Novel phenyl triazole-pyridylpiperazine derivatives were synthesized & tested against cancer cells. Compounds 15 & 18 showed potent anticancer activity comparable to doxorubicin, with favorable ADME profiles & stable EGFR binding.

## Graphical abstract

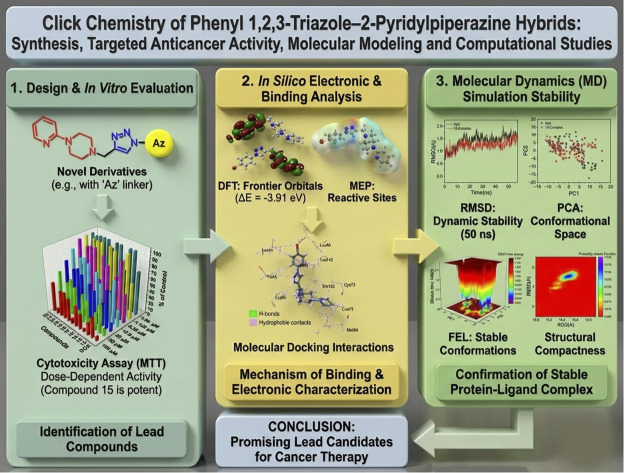

## 1. Introduction

Cancer is acknowledged as a primary cause of mortality and is regarded as one of the most significant health risks.^1^ The disruption of essential enzymes and proteins that govern cell division and growth results in cancer, which is marked by uncontrolled and accelerated cell division.^2^ Over 200 different types of cancer are identified, including those affecting the breast, lung, prostate, colon, and rectum.^3^

**Fig. 1:**
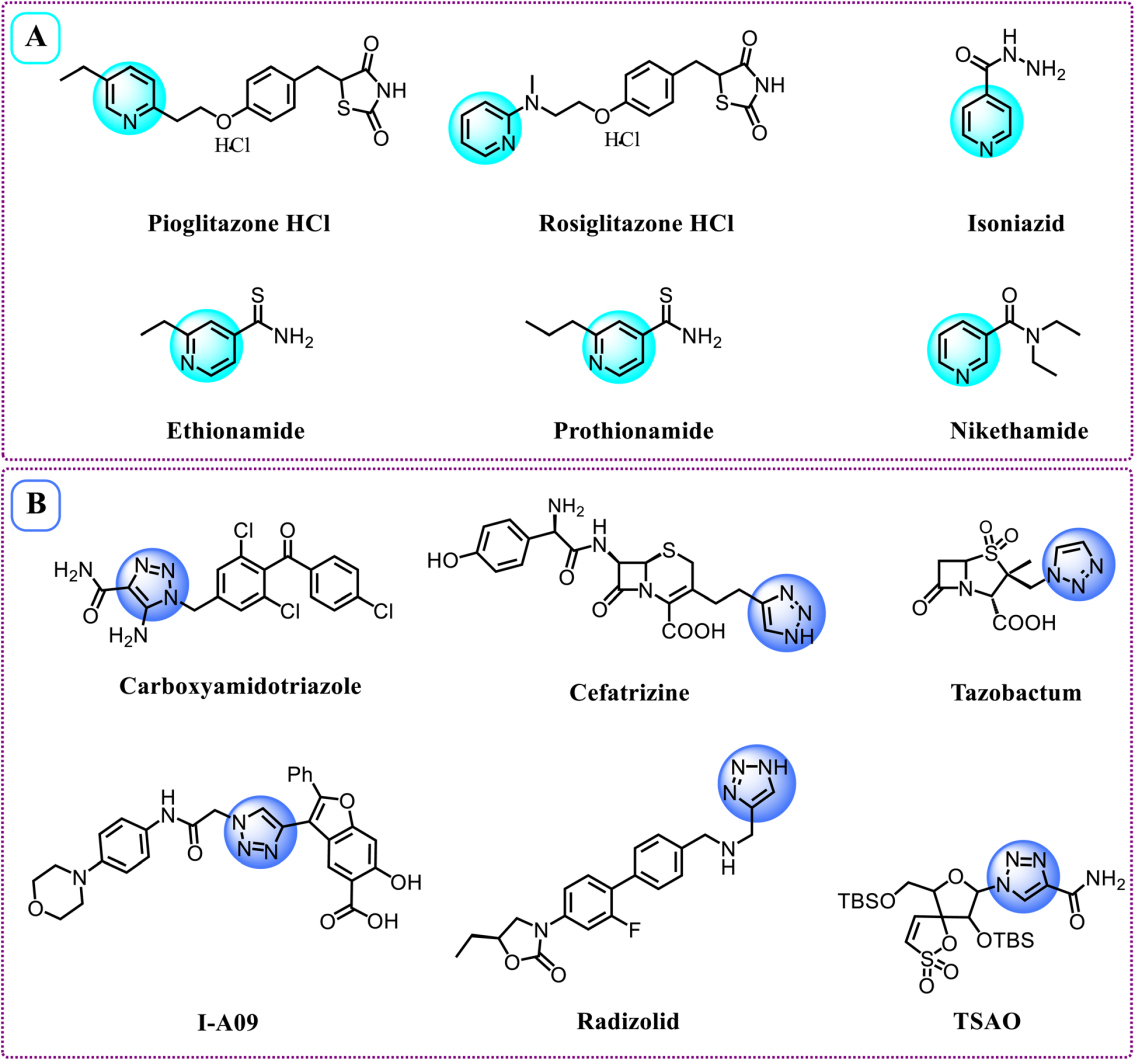
Pharmaceutical compounds featuring [A] pyridine, and [B] 1,2,3-triazoles moiety.

The World Health Organization (WHO) reported in 2020 that it was estimated that ten million people would die from this disease, accounting for one-sixth of all deaths across the globe.^4^ Estimates predict that by 2040, the total number of cancer patients globally will rise to 29.4 million, emphasizing the pressing requirement for enhanced research, superior treatment options, and effective prevention strategies.^5^ Existing medical practices continue to confront multiple challenges in cancer care. Therefore, it is essential to develop and implement new techniques for treating different types of cancer.^6^ Heterocyclic molecules are organic compounds that feature at least one atom that is not carbon, such as nitrogen, oxygen, or sulfur, within their ring structure.^7^ These compounds are significant in the realm of medical and pharmaceutical chemistry, as they serve as the core components of various biologically active molecules and pharmaceuticals.^8^ As a result of their structural diversity and their ability to engage with biological targets, heterocyclic compounds are broadly utilized in the design and development of antibiotics,^9^ anticancer agents,^10^ antivirals,^11^ and cardiovascular drugs,^12^ thus making them essential in contemporary drug discovery and therapeutic applications. Pyridine is a six-membered heterocyclic aromatic compound that consists of one nitrogen atom within its ring structure.^13^ It plays a vital role as a building block in organic and pharmaceutical chemistry due to its chemical stability and its ability to engage in different reactions.^14^ Pyridine and its derivatives are extensively utilized in numerous drugs, agrochemicals, and vitamins, playing an essential role in medicinal chemistry owing to their biological activity and robust interactions with enzymes and receptors.^15^ Currently, numerous pharmaceutical agents are commercially accessible, such as Pioglitazone HCl, Rosiglitazone HCl, Isoniazid, Ethionamide, Prothionamide, and Nikethamide (illustrated in Fig. 1A). These drug molecules share a common structural feature—a pyridine ring—which plays a critical role in their therapeutic effects. Moreover, recent drug development efforts have increasingly turned attention to 1,2,3-triazoles. This interest stems from their straightforward preparation through click chemistry techniques and their wide range of bioactive properties, as noted in ref. 16. The Cu(i)-catalyzed azide–alkyne cycloaddition (CuAAC) reaction facilitates the effective synthesis of 1,2,3-triazole heterocycles under mild conditions, providing high yields, stability, and excellent chemoselectivity.^17^ As a consequence, triazole-derived molecular systems have been extensively applied in medicine,^18^ materials science,^19^ and coordination chemistry.^20^ The 1,2,3-triazole structure is a vital component in numerous pharmaceutical products, such as Carboxyamidotriazole, Cefatrizine, Tazobactam, I-A09, Radizolid, and TSAO^21^ (Fig. 1B), and is associated with various pharmacological activities, including antibacterial,^22,23^ anticancer,^24^ antiviral,^25,26^ anti-inflammatory,^27^ and antidiabetic effects.^27^ Epidermal growth factor receptor (EGFR) is a validated therapeutic target in cancer due to its key role in regulating cell proliferation and survival, with its overexpression reported in several malignancies. Most EGFR inhibitors act by targeting the ATP-binding site of the tyrosine kinase domain, where effective inhibition requires specific pharmacophoric features, including a heterocyclic core capable of forming hydrogen bonds with the hinge region, hydrophobic moieties for pocket occupancy, and suitable electronic properties for optimal binding. Structure–activity relationship (SAR) studies have further highlighted the importance of functional group orientation and linker flexibility in enhancing activity and selectivity. Therefore, EGFR represents a rational target for the development of new anticancer agents. Recent studies have also demonstrated that hybrid molecules integrating multiple pharmacophores can improve binding affinity and biological performance.^28,29^ In this context, the incorporation of pyridine and 1,2,3-triazole moieties represents a promising strategy for designing effective EGFR inhibitors. In this research, we present the synthesis and characterization of phenyl 1,2,3-triazole–2-pyridylpiperazine derivatives utilizing a click chemistry methodology. Considering the facts outlined above, a series of novel heterocyclic compounds incorporating pyridine and 1,2,3-triazole moieties were efficiently prepared *via* the standard Cu(i)-catalyzed azide–alkyne cycloaddition (CuAAC) protocol. The cytotoxic activities of these hybrid molecules were evaluated against several cancer cell lines including colorectal carcinoma (HCT-116), liver cancer (Hep-G2), and breast adenocarcinoma (MCF-7) alongside a normal human cell line (WI-38). Additionally, molecular docking along with molecular dynamics (MD) simulations were conducted to clarify the binding modes and significant interactions of the synthesized compounds within the active site of the target receptor.

## 2. Results and discussion

### 2.1. Chemistry

**Scheme 1:**
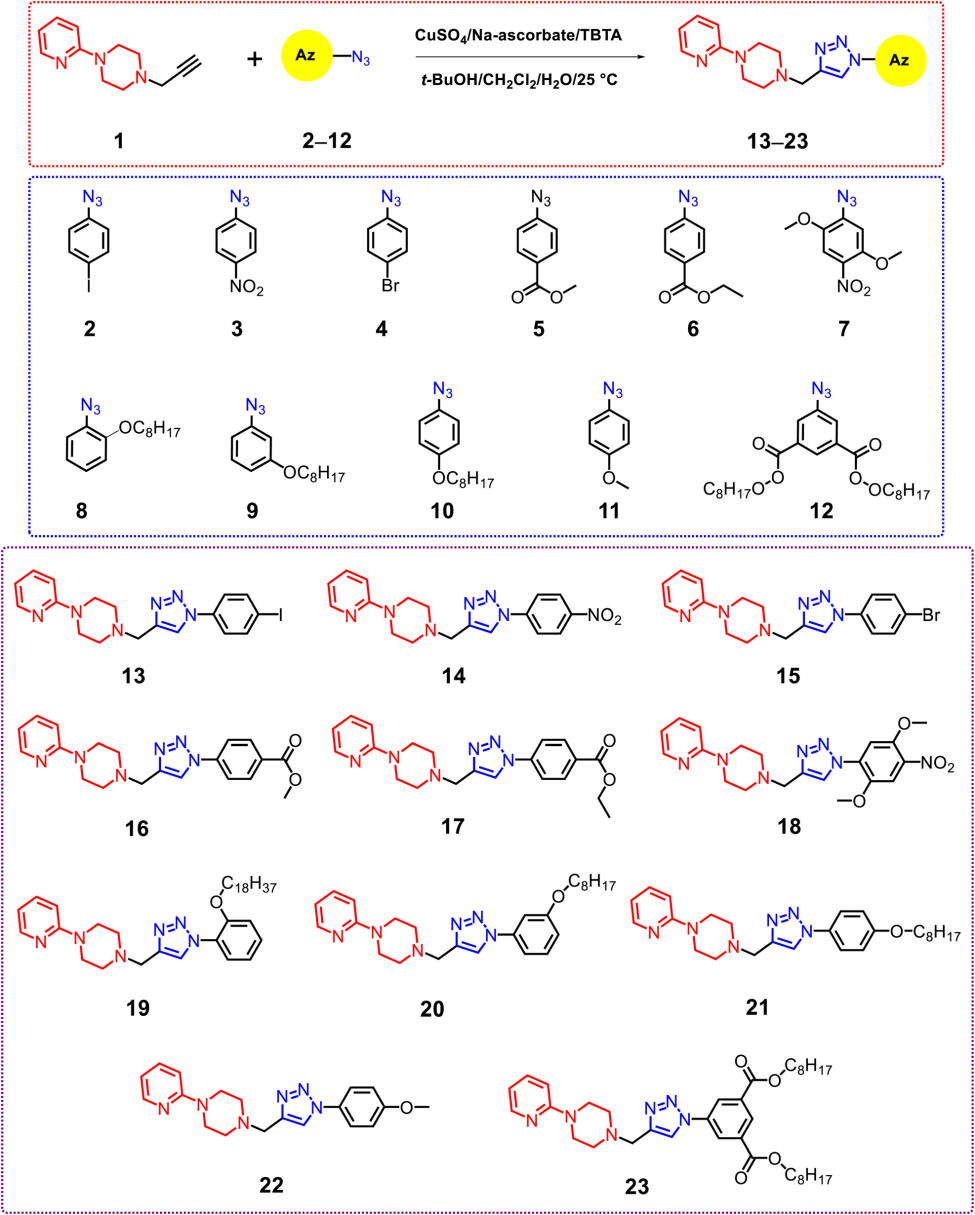
Synthetic pathway of phenyl 1,2,3-triazoles–2-pyridylpiperazine derivatives 13–23.

The synthesis of phenyl 1,2,3-triazoles–2-pyridylpiperazine derivatives 13–23, represented in Scheme 1, has been determined to be the most efficient strategy for developing the target compounds. Utilizing click chemistry, 1-(prop-2-yn-1-yl)-4-(pyridin-2-yl)piperazine 1 (ref. 30) was successfully integrated with aromatic azides 2–12,^18,31–33^ yielding a modular synthesis with impressive yields (82–93%). The Cu^I^-catalyzed 1,3-dipolar cycloaddition reaction was performed in accordance with a defined protocol,^34–36^ which entailed the *in situ* production of Cu(i) from CuSO_4_ and sodium ascorbate. Additionally, tris[(1-benzyl-1*H*-1,2,3-triazol-4-yl)methyl]amine (TBTA) was incorporated as a stabilizing ligand, and the reaction occurred within a biphasic system consisting of *tert*-butanol and methylene chloride. For instance, the ^1^H NMR spectrum of compound 16 indicated the existence of methylene groups from piperazine (2CH_2*pip*_), which appeared as a double triplet at *δ* 2.76 and 3.63. Furthermore, the methylene protons acting as a bridge linker between the acetylene and azido derivative (CH_2*linker*_) were observed at *δ* 3.90, while the singlet signal for the acetyl group (OCH_3_) was noted at *δ* = 3.96. The triazolo proton was detected at *δ* 8.20 ppm, in addition to the aromatic protons. The ^13^C NMR spectrum for compound 16 revealed the presence of methylene groups from piperazine (2CH_2pip_) at *δ* 44.91 and 52.69, a methyl carbon (CO_2_CH_3_) at *δ* 52.34, a methylene carbon (NCH_2linker_) serving as a bridge linker at *δ* 53.16, and a carbonyl carbon (CO_2_) at *δ* = 165.71. The mass spectrum of compound 16 displayed a molecular ion peak at *m*/*z* = 378.51, recognized as [M^+^], which aligns with the molecular formula C_20_H_22_N_6_O_2_. Furthermore, the structure of derivative 16 was validated by elemental analysis, as detailed in the experimental section. In another example, the ^1^H NMR spectrum of compound 21 exhibited a triplet signal at *δ* 0.89 ppm, characteristic of the CH_3_ group. The methylene (CH_2_) signals were found in the range of *δ* 1.30–1.47 ppm, linked to the long aliphatic side chain (C_8_H_17_). Methylene groups from piperazine (2CH_2*pip*_) appeared as a double triplet at *δ* 2.73 and 3.60. Additionally, the methylene protons serving as a bridge linker between the acetylene and azido derivative (CH_2*linker*_) were detected at *δ* 3.85. Also, methylene (OCH_2_) triplet signals were noted at *δ* 4.00 ppm, related to the aliphatic side chain. The CH singlet signal from the triazole ring was recorded at *δ* 8.18 ppm. The ^13^C-NMR spectrum associated with compound 21 showed signals at *δ* 14.03 and *δ* 68.27 ppm, which relate to the CH_3_ group and the OCH_2_ group present in the long aliphatic side chain (C_8_H_17_). The spectrum also highlights the presence of methylene groups from piperazine (2CH_2pip_) at *δ* 44.87 and 52.60, a methylene carbon (NCH_2linker_) that acts as a bridge linker at *δ* 53.20, and a carbonyl group (CO) at *δ* = 159.18. Additionally, the EI-mass spectrum revealed the expected molecular ion peak at *m*/*z* 449.69; [M + H^+^]. Following the successful synthesis and characterization of the target derivatives 13–23, we shifted our attention to investigating their anticancer activity.

### 2.2. Biology

#### 2.2.1. Anticancer activity

**Fig. 2:**
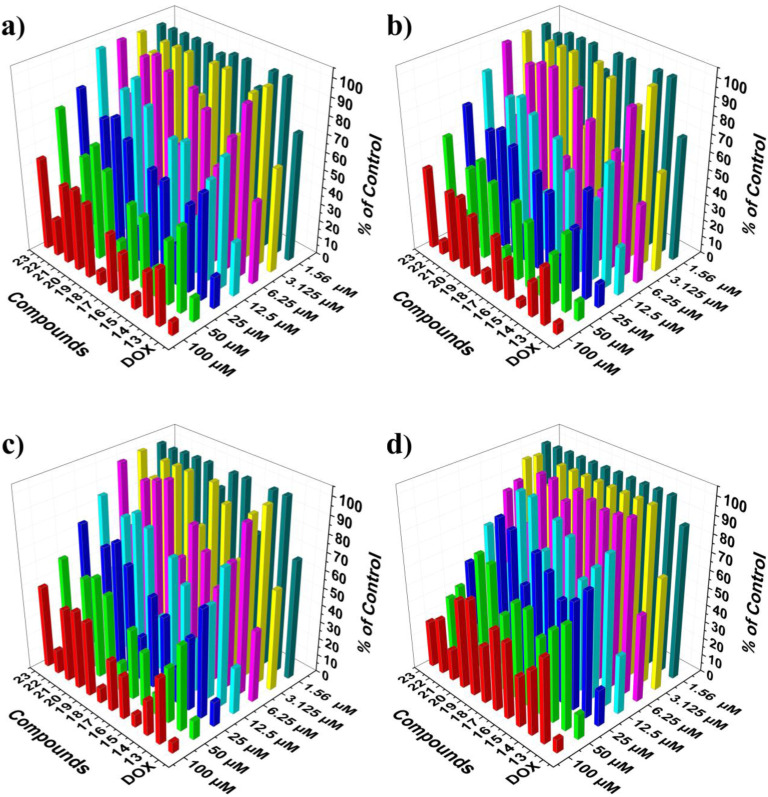
The cell viability percentage of the phenyl 1,2,3-triazoles–2-pyridylpiperazine derivatives 13–23 was measured across various cancer cell lines (a) HCT-116, (b) HePG-2, (c) MCF-7, and (d) WI38.

After the successful synthesis and identification of the novel phenyl 1,2,3-triazoles-2-pyridylpiperazine derivatives 13–23, their antiproliferative effects were assessed against three cancer cell lines: HCT-116, HePG-2, and MCF-7, along with the normal cell line WI38. The cancer cells were treated with a range of concentrations of the compounds under examination for a duration of 48 hours. The IC_50_ values, indicating the concentrations required to inhibit 50% of tumor cell proliferation, are presented in Table 1, with doxorubicin serving as the reference drug. As represented in Fig. 2a and summarized in Table 1, compounds 15 and 18 exhibited pronounced cytotoxic activity against the HCT-116 cell line, with IC_50_ values of 7.48 ± 0.6 µM and 9.19 ± 0.8 µM, respectively. Compound 22 also demonstrated considerable cytotoxic potential, recording an IC_50_ value of 18.32 ± 1.4 µM. In contrast, compounds 14, 16, 17, and 13 showed only moderate inhibitory effects, with IC_50_ values of 26.20 ± 1.7, 35.27 ± 2.2, 41.51 ± 2.5, and 46.68 ± 2.7 µM, respectively. Meanwhile, derivatives 19, 21, 20, and 23 exhibited relatively weak cytotoxic activities when compared with the reference drug, doxorubicin. In the evaluation of compounds for their effects on HepG-2 human liver cancer cells, Compounds 15, 18, 22, and 14 were found to exhibit very strong cytotoxic effects, as indicated by IC_50_ values of 3.29 ± 0.2, 6.56 ± 0.5, 9.85 ± 0.7, and 15.58 ± 1.2 µM, respectively. On the other hand, compounds 16, 17, 13, and 19 were observed to have moderate cytotoxic effects, with their IC_50_ values recorded at 24.69 ± 1.6, 39.57 ± 2.4, 43.52 ± 2.6, and 48.82 ± 2.8 µM, respectively. Furthermore, derivatives 21, 20, and 23 showed weak cytotoxic properties, with IC_50_ values of 56.03 ± 3.2, 67.49 ± 3.5, and 81.71 ± 4.1 µM, respectively (Fig. 2b and Table 1). The MCF-7 human breast cancer cell line has exhibited improved cytotoxic effects from two distinct compounds, 15 and 18, which have IC_50_ values of 5.75 ± 0.4 and 8.32 ± 0.6 µM, respectively. On the other hand, compounds 22 and 14 present marginally lower cytotoxicity, with IC_50_ values of 12.93 ± 0.9 and 19.60 ± 1.4 µM. Furthermore, compounds 16 and 17 reveal moderate cytotoxic activity, with IC_50_ values of 21.59 ± 1.7 and 33.45 ± 2.1 µM, respectively. Finally, compounds 13, 19, 20, 21, and 23 exhibit weak cytotoxic effects on MCF-7 when assessed against the reference drug, as depicted in Fig. 2c and Table 1.

**Table 1:** The anti-proliferative effects of new compounds phenyl 1,2,3-triazoles–2-pyridylpiperazine derivatives 13–23^a^

Entry	IC_50_ (µM) ± SD			
	HCT-116	HePG-2	MCF-7	WI-38
13	46.68 ± 2.7	43.52 ± 2.6	51.47 ± 2.9	84.43 ± 4.2
14	26.20 ± 1.7	15.58 ± 1.2	19.60 ± 1.4	53.09 ± 3.1
15	7.48 ± 0.6	3.29 ± 0.2	5.75 ± 0.4	37.97 ± 2.5
16	35.27 ± 2.2	24.69 ± 1.6	21.59 ± 1.7	69.81 ± 3.5
17	41.51 ± 2.5	39.57 ± 2.4	33.45 ± 2.1	76.86 ± 3.8
18	9.19 ± 0.8	6.56 ± 0.5	8.32 ± 0.6	39.94 ± 2.4
19	65.73 ± 3.5	48.82 ± 2.8	59.67 ± 3.3	>100
20	87.65 ± 4.3	67.49 ± 3.5	74.50 ± 3.8	>100
21	72.66 ± 3.8	56.03 ± 3.2	62.41 ± 3.5	16.26 ± 1.4
22	18.32 ± 1.4	9.85 ± 0.7	12.93 ± 0.9	40.87 ± 2.5
23	>100	81.71 ± 4.1	78.91 ± 3.7	28.84 ± 2.0
DOX	5.23 ± 0.3	4.50 ± 0.2	4.17 ± 0.2	6.72 ± 0.5

a IC_50_ (µM): 1–10 (very strong). 11–20 (strong). 21–50 (moderate). 51–100 (weak) and above 100 (non-cytotoxic). DOX: doxorubicin.

The selectivity of the synthesized compounds was assessed using the normal human lung fibroblast cell line WI-38. As shown in Table 1 and Fig. 2d, the most potent anticancer compounds 15 and 18 exhibited moderate cytotoxicity against WI-38 with IC_50_ values of 37.97 ± 2.5 and 39.94 ± 2.4 µM, respectively, representing substantially lower toxicity toward normal cells compared to cancer cells. This favorable selectivity profile was quantified by calculating selectivity indices (SI = IC_50_ normal/IC_50_ cancer). Compound 15 demonstrated excellent selectivity with SI values of 5.1 against HCT-116, 11.5 against HepG-2, and 6.6 against MCF-7. Similarly, compound 18 showed good selectivity with SI values of 4.3, 6.1, and 4.8, respectively. These values compare favorably with doxorubicin, which exhibited poor selectivity (SI = 1.3–1.6 across all cell lines). In contrast, compound 21 displayed strong cytotoxicity against WI-38 (IC_50_ = 16.26 ± 1.4 µM) while showing only weak antiproliferative activity against cancer cells (IC_50_ = 56.03–72.66 µM). The resulting SI values of 0.22–0.29 indicate that this compound is more toxic to normal cells than to cancer cells, rendering it unsuitable for further development. Likewise, compound 23 exhibited moderate toxicity toward WI-38 (IC_50_ = 28.84 ± 2.0 µM) with negligible anticancer activity (IC_50_ > 78 µM), also yielding SI < 0.4. These findings underscore the critical importance of evaluating compounds against normal cell lines to identify candidates with genuine therapeutic potential. The reference drug doxorubicin exhibited variable IC_50_ values across the tested cell lines (4.17–6.72 µM), which is consistent with its known differential sensitivity profiles in cancer cell lines and its inherent toxicity toward normal fibroblasts. These values fall within the expected range for doxorubicin in MTT assays and provide a valid benchmark for comparative assessment of the synthesized compounds.

### 2.3. The structure–activity relationship (SAR)

The structure–activity relationship (SAR) analysis of synthesized phenyl 1,2,3-triazole–2-pyridylpiperazine derivatives (13–23) demonstrates the critical role of substituents on the aromatic moiety in modulating antiproliferative activity. Derivatives 15 and 18 emerged as the most potent across all cancer cell lines, highlighting that specific substitution patterns enhance biological activity due to favorable electronic and hydrophobic interactions at the target binding site. Compound 22 displayed strong activity with moderately substituted aromatic groups, indicating effectiveness despite reduced potency. In contrast, compounds 14, 16, and 17 showed moderate activity, possibly due to suboptimal substituents or steric hindrance affecting target binding. Compounds 19–21 and 23 exhibited weak to negligible activity, likely due to unfavorable electronic properties or steric hindrance impairing binding affinity. Notably, compound 21 demonstrated higher toxicity toward normal cells (WI-38), indicating potential compromises in selectivity due to certain substitution patterns. Compounds 15 and 18 not only exhibited strong antiproliferative activity but also favorable selectivity indices, marking them as promising lead candidates. The SAR findings underscore the necessity for an optimal balance of electronic properties, hydrophobicity, and steric factors to enhance anticancer activity and selectivity among this compound class.

### 2.4. Molecular dynamic (MD) simulations

#### 2.4.1. Molecular dynamic and system stability

**Fig. 3:**
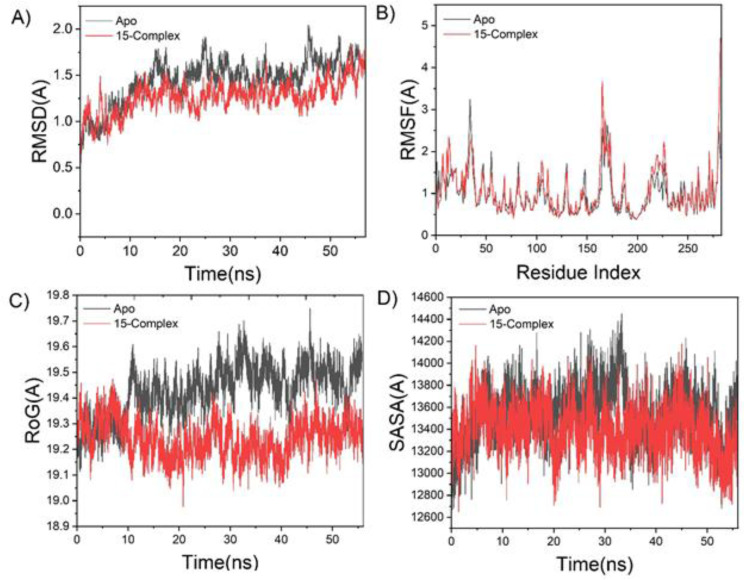
(A) Root-Mean-Square Deviation (RMSD) of the C_α_ atoms from the protein backbone. (B) Root-Mean-Square Fluctuation (RMSF) per residue for the C_α_ atoms of the protein backbone. (C) Radius of gyration (*R*_g_) of the protein's C_α_ atoms. (D) Solvent-accessible surface area (SASA) of the backbone C_α_ atoms. In all panels, the black trace represents the relative values compared to the starting minimized structure, while the red trace corresponds to the ATP-binding site of the EGFR receptor in complex with compound 15, monitored over a 60 ns simulation period.

To predict how the extracted compounds behave when bound to the protein's active site, including their interactions and stability over time, a molecular dynamic simulation was conducted.^37^ Verifying the system's stability is essential for monitoring unusual movements and avoiding artifacts that could arise during the simulation. In this research, Root-Mean-Square Deviation (RMSD) was employed to assess system stability throughout the 60 ns simulation duration. The mean RMSD values documented across all simulation frames were 1.74 ± 0.23 Å for the EGFR–Apo system and 1.51 ± 0.17 Å for the EGFR–15 complex (Fig. 3A). The results demonstrated that the system featuring the protein linked to compound 15 achieved a more stable conformation relative to the Apo system. Throughout the molecular dynamics simulation, it is essential to evaluate the changes in protein structural flexibility resulting from ligand binding to gain insights into residue behavior and their interactions with the ligand.^38^ Residue fluctuations were assessed through the Root-Mean-Square Fluctuation (RMSF) method to analyze how the binding of the inhibitor influences the target protein during the 60 ns simulation. The average RMSF values calculated were 1.10 ± 0.50 Å for EGFR–Apo and 0.89 ± 0.46 Å for the EGFR–15 complex (Fig. 3B).

These findings showed that the protein system bound to compound 15 exhibited lower residue fluctuation than the other system. The radius of gyration (*R*_g_) was calculated to assess overall system compactness and stability following ligand binding during the MD simulation. The average *R*_g_ values were 19.42 ± 0.10 Å for EGFR–Apo and 19.24 ± 0.07 Å for the EGFR–15 complex (Fig. 3C). Based on this observed behavior, compound 15 demonstrated a highly rigid structure relative to the receptor under investigation. The compactness of the hydrophobic core of the protein was further examined by evaluating the solvent-accessible surface area (SASA). This measurement quantifies the protein surface area exposed to the solvent, which is a key factor in biomolecular stability.^37^ The average SASA values were 13 539.90 Å^2^ for EGFR–Apo and 13 393.54 Å^2^ for the EGFR–15 complex (Fig. 3D). The SASA results, along with the findings of RMSD, RMSF, and *R*_g_, validated that the EGFR–15 complex stayed stable and intact within the binding site of the catalytic domain of the examined receptor.

#### 2.4.2. Hydrogen bond formation between amino acid residues and the ligand

**Fig. 4:**
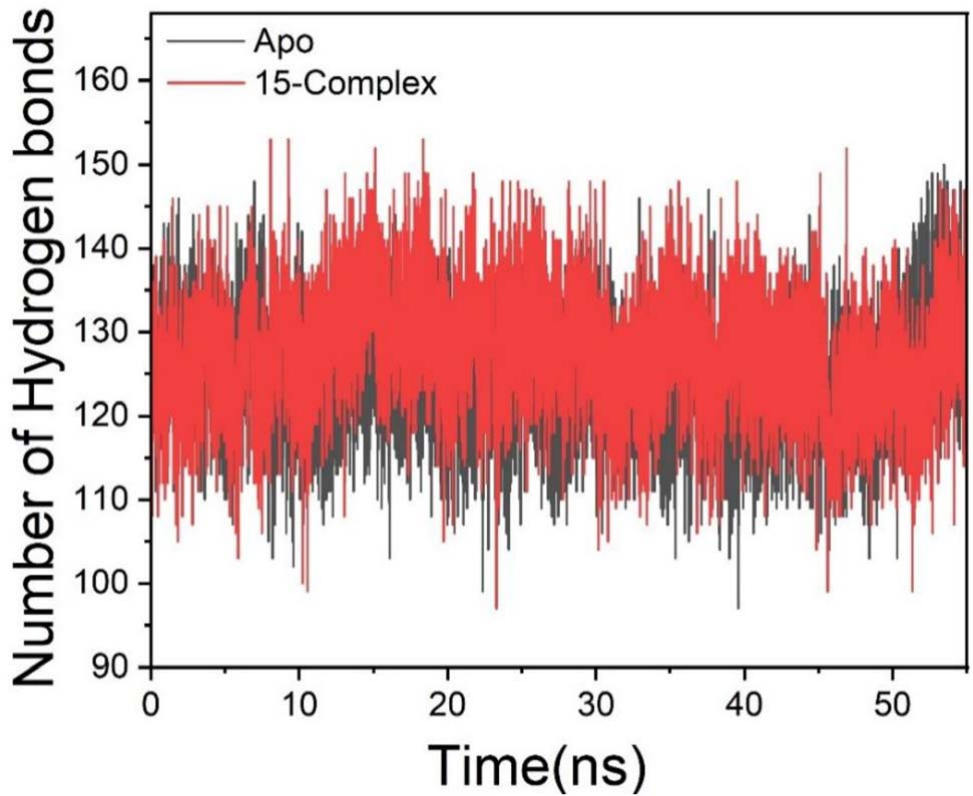
Number of hydrogen bond formation during simulation overtime between Apo, and 15-complex systems.

Hydrogen bonds (H-bonds) are pivotal in numerous chemical processes and are crucial in biological systems, especially for preserving protein structural integrity.^39^ Hydrogen bonding facilitates protein–ligand interactions, catalysis, and protein–ligand binding. Consequently, the establishment of hydrogen bonds among amino acid residues is essential for determining the conformational stability of amino acids. Consequently, we examine the development of hydrogen bonds during the simulation, as illustrated in Fig. 4. The Apo system demonstrates a reduced average hydrogen bond formation rate of 124.52 throughout simulation, in contrast to complex systems, which exhibit an average of 130.54. The reduction in hydrogen bond production results in structural destabilization and conformational instability, which subsequently impacts treatment binding.

#### 2.4.3. Binding interaction mechanism based on binding free energy calculation

One widely used approach for estimating the binding free energies between small molecules and biological macromolecules is the molecular mechanics with generalized Born and surface area solvation (MM/GBSA) method. This technique tends to provide more reliable results compared to docking scores alone.^40^ In this investigation, the MM-GBSA module included in the AMBER18 software package was used to determine binding free energies, based on snapshots derived from the system trajectories. As illustrated in Table 2, all reported energy components-apart from Δ*G*_solv_ showed substantial negative values, indicating favorable interactions.

**Table 2:** The calculated energy binding for 15 compound against the EGFR receptor^a^

Complex	Energy components (kcal mol^−1^)				
	Δ*E*_vdW_	Δ*E*_elec_	Δ*G*_gas_	Δ*G*_solv_	Δ*G*_bind_
15-Complex	−51.56 ± 0.30	−18.88 ± 0.97	−32.68 ± 0.94	27.03 ± 0.88	−59.72 ± 0.33

a Δ*E*_vdW_ = van der Waals energy; Δ*E*_ele_ = electrostatic energy; Δ*G*_solv_ = solvation free energy; Δ*G*_bind_ = calculated total binding free energy.

A detailed breakdown of the individual energy components contributing to the calculated binding free energies reveals that the interaction between compound 15 and the amino acid residues of the EGFR receptor is primarily driven by the more favorable van der Waals energy terms, as shown in Table 2.

#### 2.4.4. Identification of the critical residues responsible for ligands binding

**Fig. 5:**
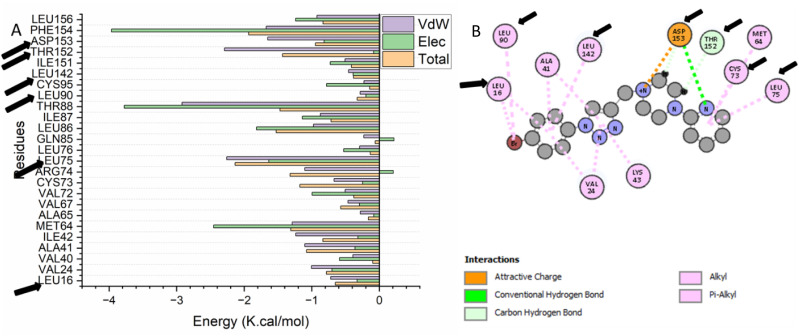
Plots depicting per-residue decomposition that reveal the energy contributions to the binding and stabilization of compound 15 within the ATP binding site of the EGFR.

To obtain a more profound understanding of the essential amino acid residues that play a crucial role in obstructing the ATP binding pocket of the EGFR receptor, the total binding energy associated with compound 15 was further broken down into contributions from individual residues within the binding site. As illustrated in Fig. 5, the most favorable energetic contribution of compound 15 to the EGFR ATP binding site comes primarily from the residues Leu 16 (−0.719 kcal mol^−1^), Val 24 (−1.00 kcal mol^−1^), Val 40 (−0.39 kcal mol^−1^), Ala 41 (−1.102 kcal mol^−1^), Ile 42 (−1.237 kcal mol^−1^), Met64 (−1.289 kcal mol^−1^), Ala 65 (−0.282 kcal mol^−1^), Val 67 (−0.465 kcal mol^−1^), Val 72 (−0.506 kcal mol^−1^), Cys 73 (−0.668 kcal mol^−1^), Arg 74 (−1.102 kcal mol^−1^), Leu 75 (−2.259 kcal mol^−1^), Leu 76 (−0.29 kcal mol^−1^), Gln 85 (−0.23 kcal mol^−1^), Leu 86 (−0.974 kcal mol^−1^), Ile 87 (−0.871 kcal mol^−1^), Thr 88 (−2.922 kcal mol^−1^), Leu 90 (−0.285 kcal mol^−1^), Cys 95 (−0.226 kcal mol^−1^), Leu 142 (−0.455 kcal mol^−1^), Ile 151 (−0.508 kcal mol^−1^), Thr 152 (−2.291 kcal mol^−1^), Asp 153 (−1.652 kcal mol^−1^), Phe 154 (−1.675 kcal mol^−1^), and Leu 156 (−0.923 kcal mol^−1^).

#### 2.4.5. Ligand–residue interaction network profiles

##### 2.4.5.1. Docked 15 compound–EGFR complex

**Fig. 6:**
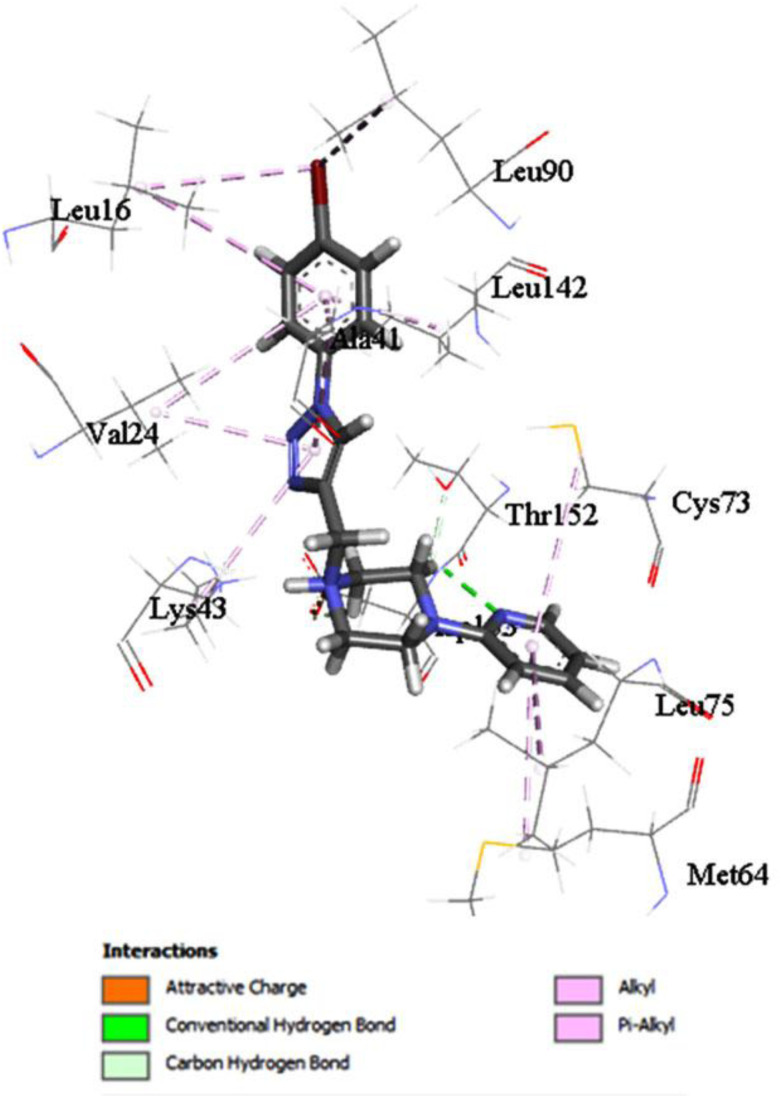
The interaction of compound 15 into the catalytic site of EGFR receptors.

Compound 15, which fits into the catalytic active site of EGFR, is shown in Fig. 6. Compound 15 has been found to form a Pi–alkyl interaction with Leu 16, Leu 90, Ala 41, Leu 142, Val 24, Lys 43, Cys 73, Met 64, and Leu 75. Finally, the pharmacophoric hot spot residue Asp 153 has formed H-bonding, attractive charge, and carbon hydrogen bonding interaction with compound 15. Noteworthy is the formation of carbon hydrogen bonding interaction between compound 15 and Thr 252.

#### 2.4.6. Principal component analysis (PCA)

**Fig. 7:**
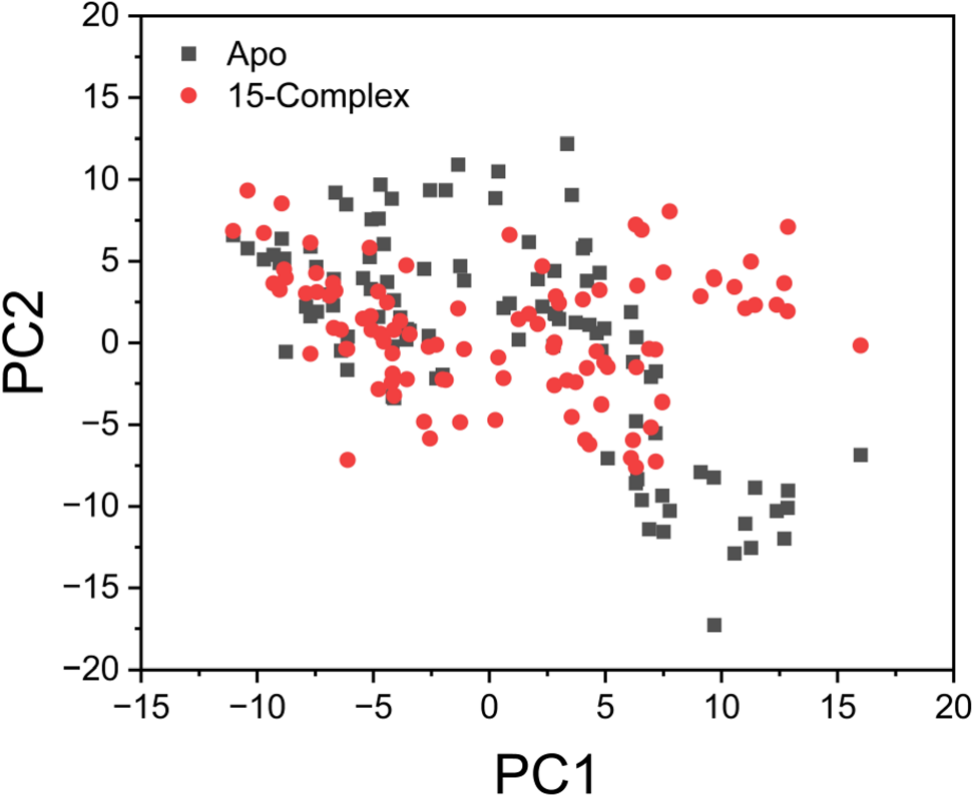
Projection of C_α_ atom motion through principal component analysis (PCA), illustrated by graphing the first two principal components (PC1 and PC2) within conformational space. The Apo form is shown in black, while compound 15 is shown in red within the EGFR receptor.

As shown in Fig. 7, the principal component analysis (PCA) plot reveals distinct and complex conformational movements along the two main components within the significant subspace. The Apo–protein system and the 15-complex system each exhibited a clear separation in their patterns of motion. As a result, the eigenvectors derived from the 60 ns molecular dynamics trajectories differed considerably between the systems, indicating variation in protein motion. The Apo system demonstrated greater atomic fluctuations compared to the 15-complex system. This suggests that ligand binding at the protein's active site within the 15-complex system induces conformational changes, which are subsequently reflected as wave-like motions in the principal components.

#### 2.4.7. Dynamics cross-correlation matrices (DCCM) analysis

**Fig. 8:**
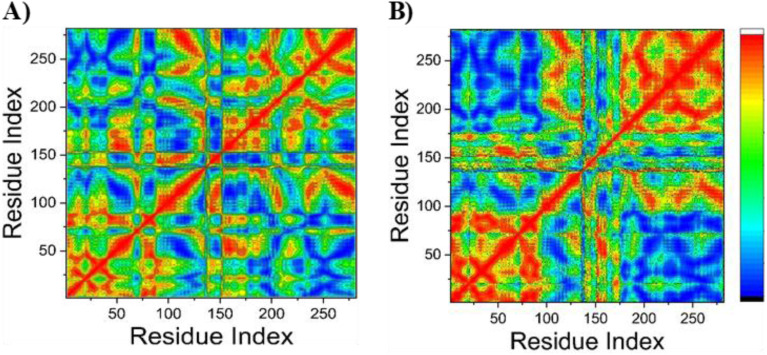
Dynamic cross-correlation matrix analyses performed for the Apo–EGFR system (A) and the EGFR protein bound to compound 15 (B). Values approaching +1 indicate a strong positive correlation between residue pairs, whereas values nearing −1 reflect a strong negative correlation or anticorrelation.

Throughout the simulation period, a dynamic cross-correlation analysis (DCCM) was performed on the C_α_ positions to investigate the existence and behavior of correlated motions (Fig. 8) and to evaluate conformational alterations in the EGFR receptor subsequent to ligand interaction. In the correlation maps, yellow-to-red regions represent strongly positive correlated motions between specific residue pairs, whereas blue-to-black regions indicate highly negative correlated motions. Across the systems analyzed in this study, overall correlated movements among residues were more prominent than anti-correlated ones. The DCCM results revealed that the binding of compound 15 to the EGFR protein imparts structural dynamics to the receptor, leading to conformational changes reflected by alterations in the associated motion patterns. As shown in Fig. 8, distinct correlated regions are observed when compound 15 binds to the EGFR protein. Notably, significant positive correlations are found in residue ranges 0–100 and 200–300 of the EGFR receptor. These segments represent the most dynamic regions of the receptor and contain the majority of hydrophobic residues within the active site.

#### 2.4.8. Free energy landscape (FEL) analysis

**Fig. 9:**
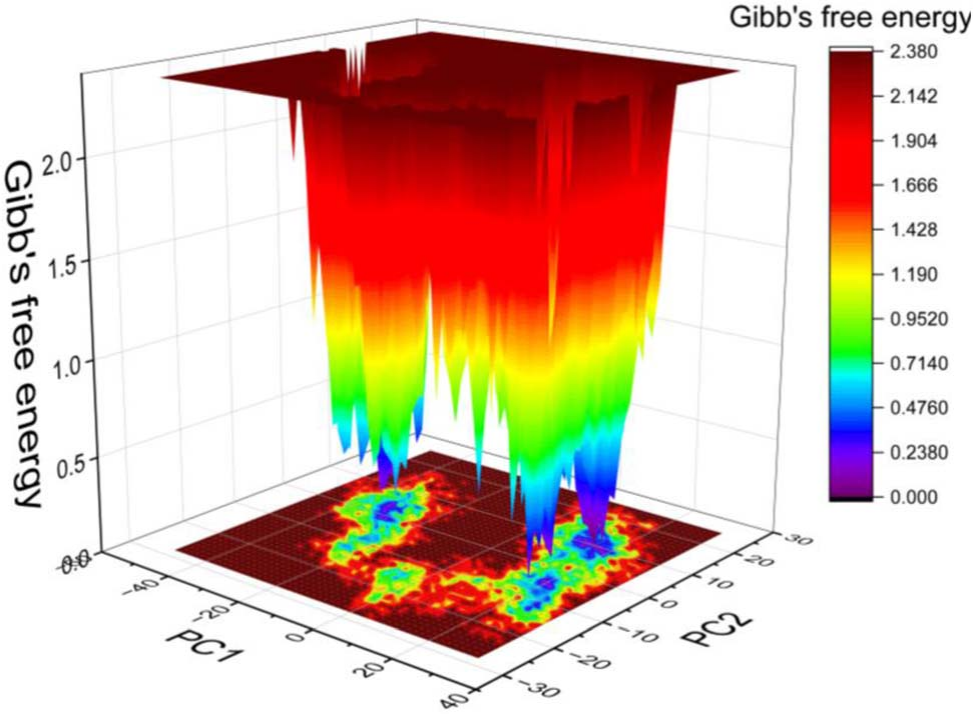
Free energy landscape (FEL) of EGFR–compound 15 complex.

As protein conformational stability correlates with lower Gibbs free energy values, a free energy landscape (FEL) utilizing principal component analysis (PCA) was created to analyze the conformational stability of the simulated complexes, as demonstrated in Fig. 9. The results of the FEL analysis showed that the simulated complex was able to attain a stable conformation, characterized by low Gibbs free energy, which is represented by the blue and violet regions in Fig. 9.

#### 2.4.9. Probability density function (PDF) analysis

**Fig. 10:**
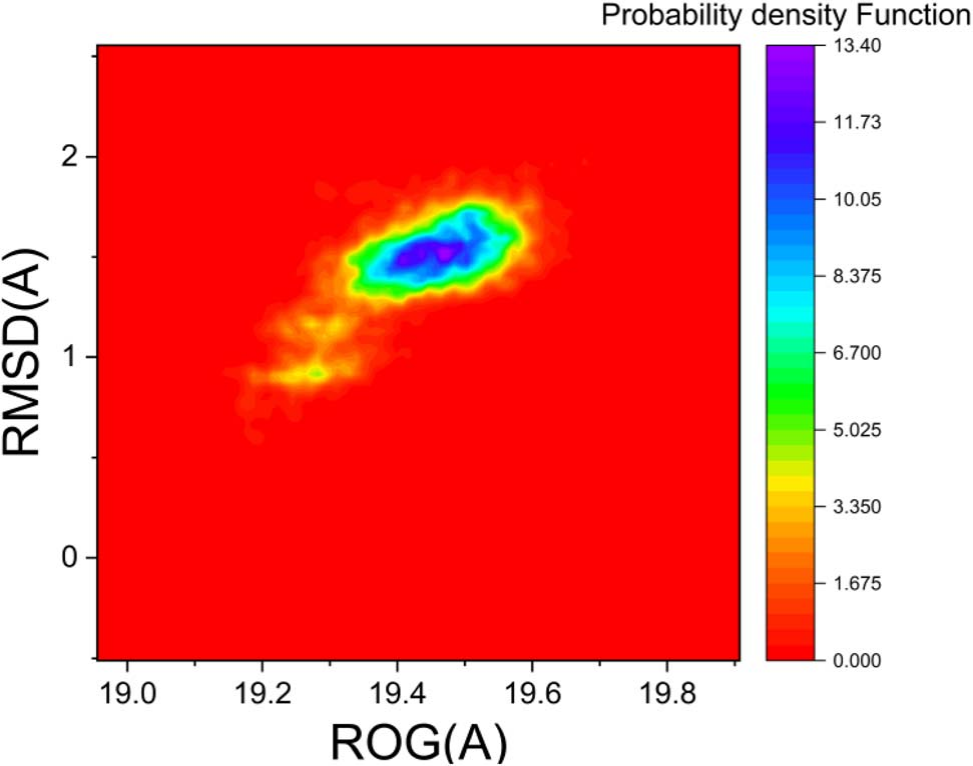
Probability density function (PDF) of EGFR–compound 15 complex the least (red) and the most (blue) populated conformations.

The probability density function (PDF) analysis, which relies on kernel density estimation (KDE), indicates the frequency of occurrence of protein trajectory states.^41^Fig. 10 presents the PDF plots based on radius of gyration (*R*_g_) and RMSD values for the EGFR–compound 15 complex. Significantly, the PDF analysis for this complex indicated that the conformations with the highest population are associated with an *R*_g_ value of 19.41 Å and an RMSD value of 1.52 Å (refer to Fig. 10).

#### 2.4.10. *In silico* drug-likeness predictions Petra/Osiris/Molinspiration (POM)

Drug-likeness prediction is an essential component of modern drug discovery, enabling the identification of compounds with favorable pharmacokinetic properties prior to experimental testing.^42^ The Lipinski Rule of Five^43^ is still the predominant standard for determining the potential for oral bioavailability, which requires that molecular weight (MW) is ≤500, calculated Log *P* (cLog *P*) is ≤5, hydrogen bond donors (HBD) are ≤5, and hydrogen bond acceptors (HBA) are ≤10. Moreover, other factors including topological polar surface area (TPSA), number of rotatable bonds (NRB), and synthetic accessibility provide further insight into drug-like behavior. Lipinski rule compliance: as presented in Tables 3 and 4, the majority of synthesized phenyl 1,2,3-triazole–2-pyridylpiperazine derivatives demonstrate favorable drug-like properties. Compounds 13–18 and 20–22 fully satisfy all Lipinski criteria, with molecular weights ranging from 350.42 to 448.60 Da and cLog *P* values between 0.013 and 4.90. All compounds exhibit zero hydrogen bond donors and acceptable hydrogen bond acceptor counts (4–8), well within Lipinski limits. TPSA values range from 50.08 to 114.36 Å^2^, with most compounds below 100 Å^2^, predicting good intestinal absorption and cell membrane permeability. Compounds with Lipinski violations: compounds 19 and 23 exceed Lipinski thresholds due to their high molecular weights (588.87 and 632.84 Da, respectively) and elevated lipophilicity (cLog *P* 8.68 and 7.17; iLog *P* 6.94 and 6.87). These properties predict poor oral bioavailability and high metabolic clearance, rendering these compounds unsuitable for oral drug development. The long aliphatic side chains present in these structures (C_18_ and C_8_ ester chains) are responsible for the observed deviations from drug-like space. Notably, the most potent anticancer compounds identified in this study 15 and 18 are both fully Lipinski-compliant, supporting their potential as orally bioavailable drug candidates with favorable absorption and permeability profiles. Solubility and Synthetic Accessibility: Aqueous solubility (log *S*) predictions range from −2.04 to −4.57 for Lipinski-compliant compounds, indicating moderate to good solubility. Synthetic accessibility scores of 3.02–3.67 for these compounds suggest facile synthesis, which is advantageous for further lead optimization. Compounds 19 and 23 show poor predicted solubility (log *S* < −6.0) and higher synthetic complexity (scores > 5.2), further supporting their deprioritization. Toxicity Risk Assessment: DataWarrior toxicity prediction models indicate that all synthesized compounds are free from mutagenic, tumorigenic, reproductive, and irritant effects (Table 3). This favorable predicted toxicity profile, combined with acceptable drug-likeness parameters for the majority of compounds, supports the therapeutic potential of this scaffold. Summary: nine of eleven synthesized compounds (82%) comply with Lipinski's rule of five and exhibit favorable predicted pharmacokinetic properties. The lead anticancer candidates 15 and 18 combine potent antiproliferative activity with excellent drug-likeness, positioning them as promising hits for further optimization. Compounds 19 and 23, while synthetically interesting, are not drug-like and should be deprioritized for pharmaceutical development.

**Table 3:** Physicochemical properties and toxicity risks of compounds 13–23 predicted using DATA Warrior

Compound	Formula	MW	cLog *P*	clog *S*	Mutagenic	Tumorigenic	Reproductive effect	Irritant
13	C_18_H_19_IN_6_	446.29	1.51	−3.08	None	None	None	None
14	C_18_H_19_N_7_O_2_	365.39	1.80	−2.85	None	None	None	None
15	C_18_H_19_BrN_6_	399.29	1.80	−2.85	None	None	None	None
16	C_21_H_26_N_6_O_2_	394.47	0.98	−2.16	None	None	None	None
17	C_21_H_24_N_6_O_2_	392.45	1.39	−2.46	None	None	None	None
18	C_20_H_23_N_7_O_4_	425.44	3.21	−2.51	None	None	None	None
19	C_36_H_56_N_6_O	588.87	8.68	−6.66	None	None	None	None
20	C_26_H_36_N_6_O	448.60	4.137	−3.96	None	None	None	None
21	C_26_H_36_N_6_O	448.60	4.13	−3.9	None	None	None	None
22	C_19_H_22_N_6_O	350.42	1.005	−2.04	None	None	None	None
23	C_36_H_52_N_6_O_4_	632.84	7.166	−6.14	None	None	None	None

**Table 4:** ADME prediction of compounds 13–23 predicted by Swiss ADME

Compound	NHD	NHA	NRB	TPSA (Å)	Log *P* (iLog *P*)	Log *S* (E SOL)	Synthetic accessibility
13	0	4	4	50.08	2.43	−4.57	3.14
14	0	6	5	95.90	2.71	−3.44	3.15
15	0	4	4	50.08	3.36	−4.30	3.02
16	0	6	6	76.38	0	−3.95	3.34
17	0	6	7	76.83	3.01	−3.69	3.28
18	0	8	7	114.36	2.75	−3.59	3.67
19	0	5	22	59.31	6.94	−9.19	5.24
20	0	5	12	59.31	4.90	−5.65	4.04
21	0	5	12	59.31	4.85	−5.85	3.93
22	0	5	5	59.31	3.36	−3.46	3.10
23	0	8	22	102.68	6.87	−8.02	5.22

#### 2.4.11. Atomic charge calculation of compounds 13–23

**Fig. 11:**
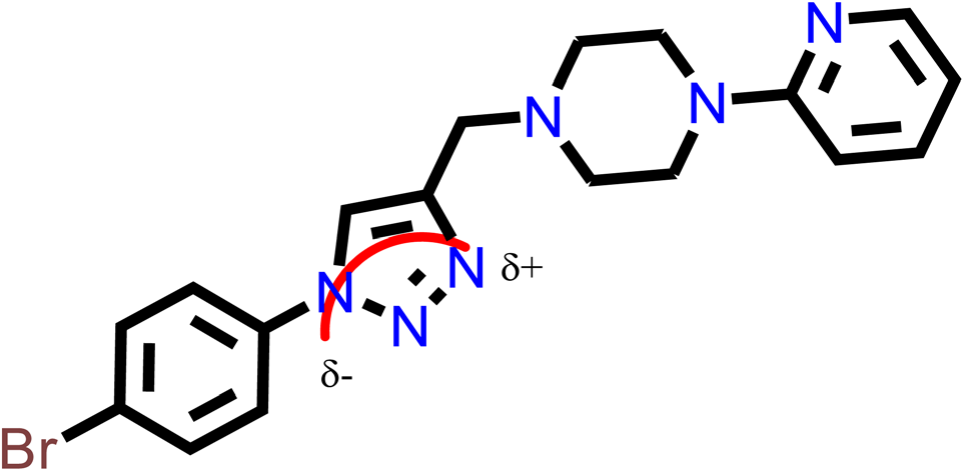
Atomic charge calculation of compound 15.

Based on the atomic charge calculation results presented in the supplementary information (Fig. S23 and S24), most of the compounds (ranging from 13 to 23) possess an anticancer pharmacophore site characterized by O^*δ*−^ and N^*δ*+^ charges. Consequently, the majority of these hits are associated with anticancer activity rather than antifungal effects. Therefore, compound 15, the most active among them, was identified as having a pharmacophore site with anticancer properties. This identification was established using the fundamental principles of Petra/Osiris/Molinspiration (POM) theory, as illustrated in Fig. 11.

#### 2.4.12. Frontier molecular orbitals (FMOs)

**Fig. 12:**
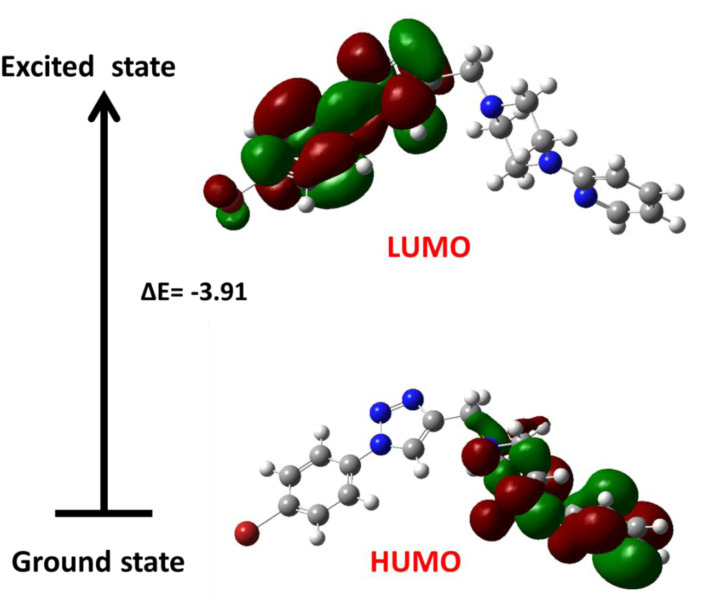
The HOMO and LUMO orbitals of compound 15.

Frontier molecular orbitals are essential for understanding kinetic behavior and chemical reactivity.^44^ A large energy gap between these orbitals indicates low reactivity and high chemical structural stability. Generally, a greater amount of energy is required for electrons to transition from the stable HOMO level to the excited LUMO level. The HOMO–LUMO energy gap (Δ*E*), along with the individual HOMO and LUMO energies and other chemical descriptors for compound 15 (the most active compound), are presented in Table 5 and illustrated in Fig. 12.

**Table 5:** Various global reactivity descriptors have been assessed for compound 15

Parameters	15
*E*_LUMO_	−0.05027
*E*_HUMO_	−0.19417
Energy band gap [*E*_HOMO_ − *E*_LUMO_] eV	3.91
Ionization potential (*I* = −*E*_HOMO_)	+0.19417
Electron affinity (*A* = −*E*_LUMO_)	+0.05027
Chemical hardness (*η*=(*I* − *A*)/2)	0.07195
Chemical softness (*ζ* = 1/2*η*)	6.9492
Electronegativity (*χ*=(*I* + *A*)/2)	0.12222
Chemical potential (*µ* = −(*I* + *A*)/2)	−0.12222
Electrophilicity index (*w* = *µ*^2^/2*η*)	0.10514
Maximum charge transfer index (Δ*N*_max_ = −*µ*/*η*)	1.7428

#### 2.4.13. Molecular electrostatic potential (MEP) analysis

**Fig. 13:**
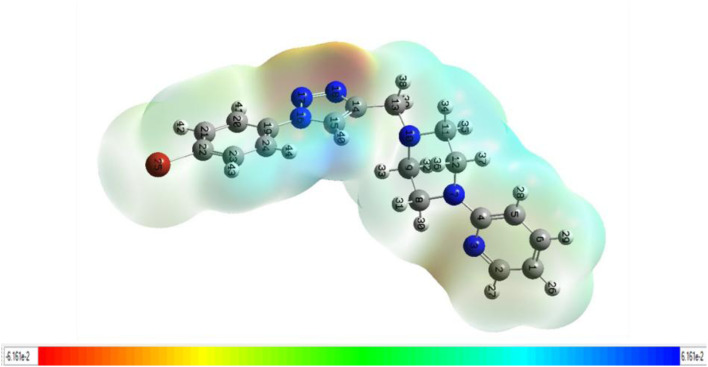
MESP map of compound 15.

Molecular electrostatic potential (MESP) analysis allows for the identification of favorable sites on ligands or protein binding regions where electrophilic or nucleophilic attacks are likely to occur.^45,46^ This strategy also contributes to visualizing the arrangement of positive and negative charges on a molecule's surface. Fig. 13 shows the MESP of compound 15, which was derived after geometry optimization using the B3LYP/3-21G basis set. MESP is useful as it can represent a molecule's size, shape, and electrostatic potential areas positive, negative, and neutral through a color gradient. This functionality aids in analyzing the relationships between molecular structures and their physicochemical properties.^47^ In the MESP map, red indicates the most negative electrostatic potential, representing regions susceptible to electrophilic attack; blue denotes the most positive potential, indicating sites favorable for nucleophilic attack; and green corresponds to neutral potential areas (Fig. 13).

## 3. Experimental of chemistry

### 3.1. Chemistry

#### 3.1.1. Materials and methods

All chemical reagents employed in the synthesis were sourced from Alfa Aesar and Sigma-Aldrich and were used without additional purification. The advancement of each reaction was tracked using thin-layer chromatography (TLC) on aluminum plates that were coated with 60 F254 silica gel, measuring 20 × 20 cm. The purification of the newly synthesized phenyl 1,2,3-triazole–2-pyridylpiperazine derivatives (compounds 13–23) was carried out *via* flash column chromatography using Silica gel 60 with a mesh size of 230–400. The nuclear magnetic resonance spectra (^1^H and ^13^C NMR) were recorded utilizing a Bruker High-Performance Digital FT-NMR spectrometer (Bruker Avance III, HD 400 MHz), which operates at 400 MHz for proton NMR and 100 MHz for carbon NMR. Electron impact mass spectrometry (EI MS) was performed on a Shimadzu GC/MS-QP2010 Plus instrument at an ionization energy of 70 eV. Elemental analyses were carried out with the aid of a Vario elemental analyzer (CHNS-932 model, made by LECO).

#### 3.1.2. General procedure of click chemistry

A mixture was prepared in a flask consisting of alkyne 1 (1 equivalent), aryl azide 2–12 (1 equivalent), sodium ascorbate (0.3 equivalent), TBTA (0.15 equivalent), along with a solvent mixture of H_2_O/^t^BuOH/CH_2_Cl_2_ in a 1/2/8 ratio, totaling 60 mL. Subsequently, 0.15 equivalents of Cu_S_O_4_·5H_2_O were introduced, and the mixture was stirred for duration of two days at room temperature. After the confirmation of alkyne 1 consumption through TLC monitoring, the mixture was diluted with CH_2_Cl_2_ and thereafter transferred into a separating funnel. The organic phase was isolated and then washed three times using an aqueous solution of disodium ethylenediaminetetraacetic acid. Following this, the aqueous phase was extracted three times with dichloromethane and subsequently washed with a saturated aqueous sodium chloride solution, totaling a volume of 60 mL. The organic phase underwent treatment with anhydrous MgSO_4_ for the purpose of drying, followed by filtration. The solvent was eliminated under vacuum conditions, facilitating the recovery of the target compound after it had been purified through column chromatography.

##### 3.1.2.1. 1-((1-(4-Iodophenyl)-1*H*-1,2,3-triazol-4-yl)methyl)-4-(pyridin-2-yl)piperazine 13

The title compound was obtained as a beige solid (92%). TLC (petroleum ether : ethyl acetate, 8 : 2) *R*_*f*_ = 0.34. ^1^H NMR (400 MHz, CDCl_3_): *δ* (ppm) = 2.81 (t, *J* = 4.54 Hz, 4H, CH_2*pip*_), 3.67 (t, *J* = 4.54 Hz, 4H, CH_2*pip*_), 3.93 (s, 2H, CH_2*linker*_), 6.63–6.67 (m, 2H, Ar*H*), 7.47–7.54 (m, 3H, Ar*H*), 7.85 (d, *J* = 8.45 Hz, 2H, Ar*H*), 8.17, 8.20 (ss, 2H, Ar*H*). ^13^C NMR (100 MHz, CDCl_3_): *δ* (ppm) = 44.64 (CH_2*pip*_), 52.48 (CH_2*pip*_), 52.96 (NCH_2*linker*_), 93.56 (I-C_Ar_), 107.07 (C_Ar_), 113.52 (C_Ar_), 121.23 (C_Ar_), 121.85 (C_Ar_), 127.86 (C_Ar_), 137.48 (C_Ar_), 138.73 (C_Ar_), 144.07 (C_Ar_), 147.79 (C_Ar_), 159.02 (C_Ar_). MS (EI, 70 eV): *m*/*z* = 446.42, [M^+^]. Elemental analysis, calcd (%) for C_18_H_19_IN_6_: C, 48.44; H, 4.29; N, 18.83. Found: C, 48.59; H, 4.17; N, 18.67.

##### 3.1.2.2. 1-((1-(4-Nitrophenyl)-1*H*-1,2,3-triazol-4-yl)methyl)-4-(pyridin-2-yl)piperazine 14

The title compound was obtained as a beige solid (90%). TLC (petroleum ether : ethyl acetate, 8 : 2) *R*_*f*_ = 0.35. ^1^H NMR (400 MHz, CDCl_3_): *δ* (ppm) = 2.74 (t, *J* = 4.81 Hz, 4H, CH_2*pip*_), 3.61 (t, *J* = 4.75 Hz, 4H, CH_2*pip*_), 3.88 (s, 2H, CH_2*linker*_), 6.61–6.66 (m, 2H, Ar*H*), 7.48 (t, *J* = 8.62 Hz, 1H, Ar*H*), 7.98 (d, *J* = 8.95 Hz, 2H, Ar*H*), 8.17, 8.19 (ss, 2H, Ar*H*), 8.40 (d, *J* = 8.96 Hz, 2H, Ar*H*). ^13^C NMR (100 MHz, CDCl_3_): *δ* (ppm) = 44.79 (CH_2*pip*_), 52.58 (CH_2*pip*_), 52.96 (NCH_2*linker*_), 106.89 (C_Ar_), 113.26 (C_Ar_), 120.14 (C_Ar_), 120.69 (C_Ar_), 125.30 (C_Ar_), 137.30 (C_Ar_), 138.41 (C_Ar_), 145.70 (C_Ar_), 146.85 (C_Ar_), 147.66 (C_Ar_), 158.07 (C_Ar_). MS (EI, 70 eV): *m*/*z* = 366.59, [M + H^+^]. Elemental analysis, calcd (%) for C_18_H_19_N_7_O_2_: C, 59.17; H, 5.24; N, 26.83. Found: C, 58.94; H, 5.30; N, 26.68.

##### 3.1.2.3. 1-((1-(4-Bromophenyl)-1*H*-1,2,3-triazol-4-yl)methyl)-4-(pyridin-2-yl)piperazine 15

The title compound was obtained as a brown solid (85%). TLC (petroleum ether : ethyl acetate, 8 : 2) *R*_*f*_ = 0.32. ^1^H NMR (400 MHz, CDCl_3_): *δ* (ppm) = 2.69 (t, *J* = 4.50 Hz, 4H, CH_2*pip*_), 3.35 (s, 2H, CH_2*linker*_), 3.58 (t, *J* = 4.52 Hz, 4H, CH_2*pip*_), 6.62–6.72 (m, 4H, Ar*H*), 7.24 (d, *J* = 8.05 Hz, 2H, Ar*H*), 7.49 (t, *J* = 7.79 Hz, 1H, Ar*H*), 8.09 (s, 1H, Ar*H*), 8.16 (d, *J* = 4.22 Hz, 1H, Ar*H*). ^13^C NMR (100 MHz, CDCl_3_): *δ* (ppm) = 44.67 (CH_2*pip*_), 46.44 (NCH_2*linker*_), 51.00 (CH_2*pip*_), 107.30 (C_Ar_), 113.30 (C_Ar_), 117.26 (C_Ar_), 127.59 (C_Ar_), 128.35 (C_Ar_), 128.70 (C_Ar_), 131.87 (C_Ar_), 137.59 (C_Ar_), 147.14 (C_Ar_), 155.95 (C_Ar_), 158.82 (C_Ar_). MS (EI, 70 eV): *m*/*z* = 400.61, [M + H^+^]. Elemental analysis, calcd (%) for C_18_H_19_BrN_6_: C, 54.14; H, 4.80; N, 21.05. Found: C, 54.23; H, 4.68; N, 21.14.

##### 3.1.2.4. Methyl 4-(4-((4-(pyridin-2-yl)piperazin-1-yl)methyl)-1*H*-1,2,3-triazol-1-yl)benzoate 16

The title compound was obtained as a beige solid (93%). TLC (petroleum ether : ethyl acetate, 8 : 2) *R*_*f*_ = 0.31. ^1^H NMR (400 MHz, CDCl_3_): *δ* (ppm) = 2.76 (t, *J* = 4.66 Hz, 4H, CH_2*pip*_), 3.63 (t, *J* = 4.71 Hz, 4H, CH_2*pip*_), 3.90 (s, 2H, CH_2*linker*_), 3.96 (s, 3H, CO_2_CH_3_), 6.63 (t, *J* = 6.28 Hz, 2H, Ar*H*), 7.34 (d, *J* = 5.67 Hz, 1H, Ar*H*), 7.45–7.51 (m, 1H, Ar*H*), 7.85 (d, *J* = 8.31 Hz, 2H, Ar*H*), 8.16–8.23 (m, 3H, Ar*H*). ^13^C NMR (100 MHz, CDCl_3_): *δ* (ppm) = 44.91 (CH_2*pip*_), 52.34 (CO_2_CH_3_), 52.69 (CH_2*pip*_), 53.16 (NCH_2*linker*_), 107.00 (C_Ar_), 113.32 (C_Ar_), 119.69 (C_Ar_), 120.76 (C_Ar_), 130.03 (C_Ar_), 131.23 (C_Ar_), 137.39 (C_Ar_), 139.95 (C_Ar_), 145.23 (C_Ar_), 147.80 (C_Ar_), 159.24 (C_Ar_), 165.71 (CO_2_). MS (EI, 70 eV): *m*/*z* = 378.51, [M^+^]. Elemental analysis, calcd (%) for C_20_H_22_N_6_O_2_: C, 63.48; H, 5.86; N, 22.21. Found: C, 63.65; H, 5.76; N, 22.34.

##### 3.1.2.5. Ethyl 4-(4-((4-(pyridin-2-yl)piperazin-1-yl)methyl)-1*H*-1,2,3-triazol-1-yl)benzoate 17

The title compound was obtained as a beige solid (91%). TLC (petroleum ether : ethyl acetate, 8 : 2) *R*_*f*_ = 0.29. ^1^H NMR (400 MHz, CDCl_3_): *δ* (ppm) = 1.43 (t, *J* = 7.12 Hz, 3H, CH_3_), 2.75 (t, *J* = 4.57 Hz, 4H, CH_2*pip*_), 3.62 (t, *J* = 4.56 Hz, 4H, CH_2*pip*_), 3.89 (s, 2H, CH_2*linker*_), 4.39 (q, *J* = 7.12 Hz, 2H, CO_2_CH_3_), 6.63 (t, *J* = 6.94 Hz, 2H, Ar*H*), 7.33 (d, *J* = 4.89 Hz, 1H, Ar*H*), 7.48 (t, *J* = 6.93 Hz, 1H, Ar*H*), 7.85 (d, *J* = 8.63 Hz, 2H, Ar*H*), 8.14–8.23 (m, 3H, Ar*H*). ^13^C NMR (100 MHz, CDCl_3_): *δ* (ppm) = 14.16 (CH_3_), 44.87 (CH_2*pip*_), 52.64 (CH_2*pip*_), 53.11 (NCH_2*linker*_), 61.27 (CO_2_CH_2_), 106.95 (C_Ar_), 113.26 (C_Ar_), 119.59 (C_Ar_), 120.73 (C_Ar_), 130.32 (C_Ar_), 131.12 (C_Ar_), 137.33 (C_Ar_), 139.83 (C_Ar_), 145.19 (C_Ar_), 147.74 (C_Ar_), 159.20 (C_Ar_), 165.23 (CO_2_). MS (EI, 70 eV): *m*/*z* = 392.48, [M^+^]. Elemental analysis, calcd (%) for C_21_H_24_N_6_O_2_: C, 64.27; H, 6.16; N, 21.41. Found: C, 64.13; H, 6.25; N, 21.49.

##### 3.1.2.6. 1-((1-(2,5-Dimethoxy-4-nitrophenyl)-1*H*-1,2,3-triazol-4-yl)methyl)-4-(pyridin-2-yl)piperazine 18

The title compound was obtained as a brown solid (84%). TLC (petroleum ether : ethyl acetate, 8 : 2) *R*_*f*_ = 0.27. ^1^H NMR (400 MHz, CDCl_3_): *δ* (ppm) = 2.69 (t, *J* = 7.29 Hz, 4H, CH_2*pip*_), 3.35 (s, 2H, CH_2*linker*_), 3.58 (t, *J* = 4.21 Hz, 4H, CH_2*pip*_), 3.95 (s, 3H, OCH_3_), 4.00 (s, 3H, OCH_3_), 6.58–6.65 (m, 2H, Ar*H*), 7.32 (s, 1H, Ar*H*), 7.46 (t, *J* = 7.82 Hz, 1H, Ar*H*), 7.66 (s, 1H, Ar*H*), 8.17 (d, *J* = 4.50 Hz, 2H, Ar*H*), 8.38 (s, 1H, Ar*H*). ^13^C NMR (100 MHz, CDCl_3_): *δ* (ppm) = 44.88 (CH_2*pip*_), 51.49 (CH_2*pip*_), 52.44 (NCH_2*linker*_), 56.78 (OCH_3_), 57.14 (OCH_3_), 106.94 (C_Ar_), 109.92 (C_Ar_), 110.15 (C_Ar_), 113.20 (C_Ar_), 123.67 (C_Ar_), 127.80 (C_Ar_), 128.47 (C_Ar_), 128.88 (C_Ar_), 134.54 (C_Ar_), 134.54 (C_Ar_), 137.31 (C_Ar_), 143.88 (C_Ar_), 147.72 (C_Ar_), 159.20 (C_Ar_). MS (EI, 70 eV): *m*/*z* = 426.50, [M + H^+^]. Elemental analysis, calcd (%) for C_20_H_23_N_7_O_4_: C, 56.46; H, 5.45; N, 23.05. Found: C, 56.25; H, 5.39; N, 23.13.

##### 3.1.2.7. 1-((1-(2-(Octadecyloxy)phenyl)-1*H*-1,2,3-triazol-4-yl)methyl)-4-(pyridin-2-yl)piperazine 19

The title compound was obtained as a solid beige (87%). TLC (petroleum ether : ethyl acetate, 8 : 2) *R*_*f*_ = 0.24. ^1^H NMR (400 MHz, CDCl_3_): *δ* (ppm) = 0.89 (t, *J* = 6.87 Hz, 3H, CH_3_), 1.12–1.43 (m, 30H, 15CH_2_), 1.75–1.77 (m, 2H, CH_2_), 2.82 (s, 4H, CH_2*pip*_), 3.68 (s, 4H, CH_2*pip*_), 3.97 (s, 2H, CH_2*linker*_), 4.05 (t, *J* = 6.15 Hz, 2H, OCH_2_), 6.63 (d, *J* = 8.56 Hz, 1H, Ar*H*), 7.09 (t, *J* = 8.51 Hz, 1H, Ar*H*), 7.34–7.40 (m, 4H, Ar*H*), 7.79 (d, *J* = 7.86 Hz, 1H, Ar*H*), 8.19 (d, *J* = 4.46 Hz, 1H, Ar*H*), 8.26 (s, 1H, Ar*H*). ^13^C NMR (100 MHz, CDCl_3_): *δ* (ppm) = 14.04 (CH_3_), 22.59, 25.90, 28.94, 29.19, 29.27, 29.60, 31.83 (CH_2_), 44.91 (CH_2*pip*_), 52.48 (CH_2*pip*_), 53.23 (NCH_2*linker*_), 68.89 (OCH_2_), 106.94 (C_Ar_), 113.02 (C_Ar_), 113.25 (C_Ar_), 120.94 (C_Ar_), 125.07 (C_Ar_), 125.23 (C_Ar_), 128.95 (C_Ar_), 129.82 (C_Ar_), 137.32 (C_Ar_), 142.87 (C_Ar_), 147.81 (C_Ar_), 150.41 (CO), 159.29 (C_Ar_). MS (EI, 70 eV): *m*/*z* = 589.16, [M^+^]. Elemental analysis, calcd (%) for C_36_H_56_N_6_O: C, 73.43; H, 9.59; N, 14.27. Found: C, 73.29; H, 9.63; N, 14.38.

##### 3.1.2.8. 1-((1-(3-(Octyloxy)phenyl)-1*H*-1,2,3-triazol-4-yl)methyl)-4-(pyridin-2-yl)piperazine 20

The title compound was obtained as a brown solid (86%). TLC (petroleum ether : ethyl acetate, 8 : 2) *R*_*f*_ = 0.25. ^1^H NMR (400 MHz, CDCl_3_): *δ* (ppm) = 0.87 (t, *J* = 6.64 Hz, 3H, CH_3_), 1.25–1.44 (m, 10H, 5CH_2_), 1.69–1.82 (m, 2H, CH_2_), 2.66 (s, 4H, CH_2*pip*_), 3.55 (s, 4H, CH_2*pip*_), 3.79 (s, 2H, CH_2*linker*_), 3.99 (t, *J* = 6.42 Hz, 2H, OCH_2_), 6.55–6.61 (m, 2H, Ar*H*), 6.85 (s, 1H, Ar*H*), 6.90 (d, *J* = 7.60 Hz, 1H, Ar*H*), 7.21 (d, *J* = 7.52 Hz, 2H, Ar*H*), 7.42 (t, *J* = 7.24 Hz, 1H, Ar*H*), 7.96 (s, 1H, Ar*H*), 8.15 (d, *J* = 4.65 Hz, 1H, Ar*H*). ^13^C NMR (100 MHz, CDCl_3_): *δ* (ppm) = 13.92 (CH_3_), 22.46, 25.81, 28.95, 29.03, 29.13, 29.51, 31.60 (CH_2_), 44.88 (CH_2*pip*_), 52.59 (CH_2*pip*_), 53.16 (NCH_2*linker*_), 68.24 (OCH_2_), 106.88 (C_Ar_), 111.87 (C_Ar_), 114.80 (C_Ar_), 120.36 (C_Ar_), 120.83 (C_Ar_), 129.93 (C_Ar_), 130.24 (C_Ar_), 137.23 (C_Ar_), 137.85 (C_Ar_), 144.64 (C_Ar_), 147.71 (C_Ar_), 159.71 (CO), 159.97 (C_Ar_). MS (EI, 70 eV): *m*/*z* = 448.73, [M^+^]. Elemental analysis, calcd (%) for C_26_H_36_N_6_O: C, 69.61; H, 8.09; N, 18.73. Found: C, 69.47; H, 8.15; N, 18.65.

##### 3.1.2.9. 1-((1-(4-(Octyloxy)phenyl)-1*H*-1,2,3-triazol-4-yl)methyl)-4-(pyridin-2-yl)piperazine 21

The title compound was obtained as a pale brown solid (88%). TLC (petroleum ether : ethyl acetate, 8 : 2) *R*_*f*_ = 0.25. ^1^H NMR (400 MHz, CDCl_3_): *δ* (ppm) = 0.89 (t, *J* = 6.59 Hz, 3H, CH_3_), 1.30–1.47 (m, 10H, 5CH_2_), 1.74–1.86 (m, 2H, CH_2_), 2.73 (t, *J* = 4.89 Hz, 4H, CH_2*pip*_), 3.60 (t, *J* = 4.88 Hz, 4H, CH_2*pip*_), 3.85 (s, 2H, CH_2*linker*_), 4.00 (t, *J* = 6.33 Hz, 2H, OCH_2_), 6.60–6.65 (m, 2H, Ar*H*), 6.99 (d, *J* = 8.97 Hz, 2H, Ar*H*), 7.47 (t, *J* = 6.89 Hz, 1H, Ar*H*), 7.61 (d, *J* = 8.95 Hz, 2H, Ar*H*), 8.18 (s, 1H, Ar*H*), 8.19 (d, *J* = 4.81 Hz, 1H, Ar*H*). ^13^C NMR (100 MHz, CDCl_3_): *δ* (ppm) = 13.97 (CH_3_), 22.49, 25.84, 28.99, 29.06, 29.17, 31.63 (CH_2_), 44.87 (CH_2*pip*_), 52.60 (CH_2*pip*_), 53.20 (NCH_2*linker*_), 68.27 (OCH_2_), 106.93 (C_Ar_), 113.20 (C_Ar_), 115.08 (C_Ar_), 120.00 (C_Ar_), 121.85 (C_Ar_), 128.88 (C_Ar_), 137.30 (C_Ar_), 144.38 (C_Ar_), 147.73 (C_Ar_), 159.18 (CO), 159.29 (C_Ar_). MS (EI, 70 eV): *m*/*z* = 449.69, [M + H^+^]. Elemental analysis, calcd (%) for C_26_H_36_N_6_O: C, 69.61; H, 8.09; N, 18.73. Found: C, 69.73; H, 7.95; N, 18.78.

##### 3.1.2.10. 1-((1-(4-Methoxyphenyl)-1*H*-1,2,3-triazol-4-yl)methyl)-4-(pyridin-2-yl)piperazine 22

The title compound was obtained as a deep brown solid (89%). TLC (petroleum ether : ethyl acetate, 8 : 2) *R*_*f*_ = 0.30. ^1^H NMR (400 MHz, CDCl_3_): *δ* (ppm) = 2.74 (s, 4H, CH_2*pip*_), 3.59 (t, *J* = 4.96 Hz, 4H, CH_2*pip*_), 3.71 (s, 2H, CH_2*linker*_), 3.86 (s, 2H, OCH_3_), 6.60–6.65 (m, 2H, Ar*H*), 7.00 (d, *J* = 8.88 Hz, 2H, Ar*H*), 7.47 (t, *J* = 6.99 Hz, 1H, Ar*H*), 7.62 (d, *J* = 8.93 Hz, 2H, Ar*H*), 7.97 (s, 1H, Ar*H*), 8.18 (d, *J* = 4.37 Hz, 1H, Ar*H*). ^13^C NMR (100 MHz, CDCl_3_): *δ* (ppm) = 44.44 (CH_2*pip*_), 52.13 (CH_2*pip*_), 53.41 (NCH_2*linker*_), 55.04 (OCH_3_), 106.59 (C_Ar_), 112.81 (C_Ar_), 114.20 (C_Ar_), 121.41 (C_Ar_), 127.37 (C_Ar_), 128.46 (C_Ar_), 134.37 (CAr), 136.94 (CAr), 147.30 (C_Ar_), 158.80 (C_Ar_), 159.13 (CO). MS (EI, 70 eV): *m*/*z* = 351.58, [M + H^+^]. Elemental analysis, calcd (%) for C_19_H_22_N_6_O: C, 65.12; H, 6.33; N, 23.98. Found: C, 64.94; H, 6.40; N, 23.87.

##### 3.1.2.11. Dioctyl 5-(4-((4-(pyridin-2-yl)piperazin-1-yl)methyl)-1*H*-1,2,3-triazol-1-yl)isophthalate 23

The title compound was obtained as a beige solid (82%). TLC (petroleum ether : ethyl acetate, 8 : 2) *R*_*f*_ = 0.28. ^1^H NMR (400 MHz, CDCl_3_): *δ* (ppm) = 0.80 (t, *J* = 6.74 Hz, 6H, CH_3_), 1.21–1.37 (m, 20H, 10CH_2_), 1.68–1.78 (m, 4H, CH_2_), 2.61 (d, *J* = 4.14 Hz, 4H, CH_2*pip*_), 3.48 (d, *J* = 4.37 Hz, 4H, CH_2*pip*_), 3.77 (s, 2H, CH_2*linker*_), 4.31 (s, 4H, OCH_2_), 6.49–6.65 (m, 2H, Ar*H*), 7.34 (t, *J* = 4.36 Hz, 1H, Ar*H*), 8.08 (d, *J* = 5.16 Hz, 1H, Ar*H*), 8.10 (s, 1H, Ar*H*), 8.54 (s, 2H, Ar*H*), 8.63 (s, 1H, Ar*H*). ^13^C NMR (100 MHz, CDCl_3_): *δ* (ppm) = 13.79 (CH_3_), 22.32, 25.65, 28.34, 28.86, 28.91, 31.46 (CH_2_), 44.74 (CH_2*pip*_), 52.50 (CH_2*pip*_), 52.97 (NCH_2*linker*_), 65.80 (CO_2_CH_2_), 106.74 (C_Ar_), 113.01 (C_Ar_), 120.77 (C_Ar_), 124.52 (C_Ar_), 128.70 (C_Ar_), 129.81 (C_Ar_), 132.47 (C_Ar_), 137.10 (C_Ar_), 145.28 (C_Ar_), 147.56 (C_Ar_), 159.04 (C_Ar_), 164.25 (CO_2_). MS (EI, 70 eV): *m*/*z* = 633.93, [M + H^+^]. Elemental analysis, calcd (%) for C_36_H_52_N_6_O_4_: C, 68.33; H, 8.28; N, 13.28. Found: C, 68.46; H, 8.35; N, 13.19.

### 3.2. Cytotoxicity assay

#### 3.2.1. Materials

Chemicals used in this study consisted of RPMI-1640 medium, MTT, and DMSO, all sourced from Sigma Co., located in St. Louis, USA. The cell lines utilized included HCT-116, HepG-2, MCF-7, and WI38, all obtained from ATCC *via* VACSERA in Cairo, Egypt.

#### 3.2.2. Procedure

An investigation was performed to evaluate the inhibitory effects of phenyl 1,2,3-triazole–2-pyridylpiperazine hybrids 13–23 on the growth of cancer cell lines *via* the MTT assay. This colorimetric assay is centered on mitochondrial succinate dehydrogenase, which modifies yellow MTT into a purple formazan in active cells.^48,49^ The cancer cell lines were grown in RPMI-1640 medium enriched with 10% fetal bovine serum, penicillin (100 units/mL), and streptomycin (100 µg mL^−1^) at a temperature of 37 °C with 5% CO_2_. All experiments were conducted in triplicate (*n* = 3). Subsequently, the cells were seeded into a 96-well plate at a density of 1.0 × 10^4^ cells per well and allowed to incubate for two days before being treated with the dendrimers at different concentrations for duration of 48 hours. After the treatment was completed, 20 µL of MTT solution (5 mg mL^−1^) was introduced, and the cells were incubated for a further 4 hours. To achieve the dissolution of formazan, 100 µL of DMSO was applied, and the colorimetric assay was carried out at an absorbance of 570 nm employing the EXL 800 plate reader (USA). The relative cell viability (%) was determined by the following equation: Cell viability (%) = A at 570 nm of treated samples/A at 570 nm of untreated samples × 100.

### 3.3. Molecular modeling and computational studies

#### 3.3.1. System preparation and molecular docking

The three-dimensional structure of the Epidermal Growth Factor Receptor (EGFR) was obtained from the Protein Data Bank under accession code 4HJO^50^ and was prepared using UCSF Chimera software.^51^ The pH was adjusted and optimized to 7.5 through the PROPKA algorithm.^52^ The two-dimensional structures of the synthesized compounds were drawn with ChemBioDraw Ultra 12.1.^53^ Energy minimization of these 2D structures was carried out using the steepest descent algorithm and the MMFF94 force field within the Avogadro software package.^54^ Prior to molecular docking, hydrogen atoms were removed from the protein structure using UCSF Chimera.^51^

#### 3.3.2. Molecular docking

Molecular docking calculations were performed using AutoDock Vina,^55^ with Gasteiger partial charges assigned to atoms prior to docking.^56^ The preparation of AutoDock atom types was carried out using the graphical interface of AutoDock within MGL Tools.^57^ A grid box centered at coordinates *x* = 26.19 Å, *y* = 14.05 Å, and *z* = 0.17 Å, with dimensions of 20 Å × 20 Å × 20 Å, defined the search space, and an exhaustiveness setting of 8 was applied. Conformations resulting from docking were generated using the Lamarckian genetic algorithm^58^ and subsequently ranked from highest to lowest based on their docking energy scores.

#### 3.3.3. Molecular dynamic (MD) simulations

##### 3.3.3.1 Molecular dynamics (MD) simulation protocol

Molecular dynamics (MD) represents a valuable computational technique for examining how atoms and molecules move within biological systems. Such simulations reveal time-dependent behaviors, including structural rearrangements and interaction patterns that are difficult to observe directly in experiments.^59^ For the current work, all MD simulations were performed using the GPU-accelerated PMEMD module included in the AMBER 18 software package.^60^

Before initiating the simulations, the ANTECHAMBER module^61^ was used to assign partial atomic charges to each ligand according to the General Amber Force Field (GAFF). Each system was then built with the LEaP tool found in AMBER 18. The protein–ligand complex was placed in an orthorhombic simulation box filled with TIP3P water molecules, ensuring that the distance from any solute atom to the box wall was at least 10 Å. Electrical neutrality was achieved by introducing Na^+^ and Cl^−^ counterions wherever required.

The energy minimization procedure comprised two sequential phases. Initially, a restrained minimization was run for 2000 steps with a harmonic force constant of 500 kcal mol^−1^·Å^−2^ applied to the solute atoms. This was followed by 1000 steps of unrestrained minimization using the conjugate gradient algorithm. After minimization, the system underwent gradual warming from 0 K to 300 K over 500 picoseconds under constant-volume (NVT) conditions. During this heating phase, a restraint of 10 kcal mol^−1^·Å^−2^ was kept on the solute atoms, and temperature regulation was provided by a Langevin thermostat operating at a collision frequency of 1 ps^−1^. Next, a 500 ps equilibration phase was performed at 300 K under constant-pressure (NPT) conditions at 1 bar, controlled by the Berendsen barostat.^62^ The final production runs lasted 60 ns within the NPT ensemble at 300 K and 1 bar, again relying on the Langevin thermostat (collision frequency = 1 ps^−1^) and the Berendsen barostat (pressure relaxation time = 2 ps). The SHAKE algorithm was applied to constrain all bonds involving hydrogen atoms, allowing a timestep of 2 femtoseconds. All simulations employed the SPFP precision model and randomized seeding. One important caveat is that the residue numbers cited in the MD analyses originate from the topology file generated by AMBER's tleap; therefore, they may differ from the standard residue numbering in the PDB entry 3ERT.

##### 3.3.3.2 Post-MD analysis

Trajectories were saved at intervals of 1 ps throughout the MD simulations. Subsequent trajectory analyses were carried out using the CPPTRAJ module^63^ of the AMBER18 suite. Graphical representations and visualizations were created using the Origin data analysis software^64^ and Chimera.^51^

#### 3.3.4. Thermodynamic calculation

Computational methods based on continuum solvation models, such as the Poisson–Boltzmann or generalized Born approaches combined with surface area terms (commonly referred to as MM/PBSA and MM/GBSA), have been shown to be effective tools for estimating how strongly ligands bind to proteins.^65^ When these techniques are applied to simulation data of protein–ligand complexes, both MM/GBSA and MM/PBSA provide thermodynamically rigorous binding free energies consistent with the chosen force field parameters. In this study, the binding free energy was computed by averaging over 600 frames taken at regular intervals from the entire 60 ns simulation trajectory. The change in binding free energy (Δ*G*) for each species involved—namely the protein–ligand complex, the unbound ligand, and the unbound receptor—can be described by the following formula:^66^

1 Δ*G*_bind_ = *G*_complex_ − *G*_receptor_ − *G*_ligand_

2 Δ*G*_bind_ = *E*_gas_ + *G*_sol_ − *TS*

3 *E*_gas_ = *E*_int_ + *E*_vdW_ + *E*_ele_

4 *G*_sol_ = *G*_GB_ + *G*_SA_

5 *G*_SA_ = *γ*SASA

Within this computational approach, the symbols *E*_gas_, *E*_int_, *E*_ele_, and *E*_vdW_ stand for the gas-phase energy, internal energy, electrostatic (Coulomb) energy, and van der Waals energy, in that order. The value of *E*_gas_ came directly from the parameters of the FF14SB force field. The free energy associated with solvation, denoted *G*_sol_, was obtained by summing the contributions from polar (GGB) and non-polar (GSA) states. To compute the non-polar solvation term GSA, the solvent-accessible surface area (SASA)^67^ was employed with a water probe radius set to 1.4 Å. For the polar part GGB, the generalized Born (GB) equation was solved. In the thermodynamic expressions, *S* represents the total entropy of the solute and *T* is the absolute temperature. Lastly, the per-residue contribution to the total binding free energy was estimated using the MM/GBSA methodology as implemented within the AMBER 18 software package.

#### 3.3.5. Principal component analysis

Principal component analysis (PCA) is a statistical technique that operates in multiple dimensions, utilized to methodically decrease the dimensionality necessary for characterizing protein dynamics.^68^ It achieves this through a decomposition process that sorts observed motions from the largest spatial scale down to the smallest. By extracting different conformational modes of the protein complex during dynamic simulations, PCA can define atomic displacements and conformational changes. Additionally, PCA allows for the determination of both the direction of motion (eigenvectors) and the magnitude of motion (eigenvalues) within a biological system. In the present study, solvent molecules and ions were first removed from the 60 ns MD trajectories using the CPPTRAJ module in Amber18 before PCA processing. PCA was then conducted on the C_α_ atoms using 1000 snapshots taken at 100 ps time intervals, employing in-house scripts. The first two principal components (PC1 and PC2) were calculated, and 2 × 2 covariance matrices were generated from the Cartesian coordinates of the C_α_ atoms. PC1 and PC2 correspond to the first two eigenvectors of the covariance matrix. The final PCA plot was constructed using Origin software.

#### 3.3.6. DCCM analysis

Dynamic cross-correlation analysis was employed to examine the fluctuations and movements of the alpha carbon backbone atoms.^69^ The cross-correlation matrix elements, denoted as Cij between the C_α_ atoms of residues *i* and *j* within the protein, can be calculated using structural data extracted from MD trajectories according to the following equation:^70^6
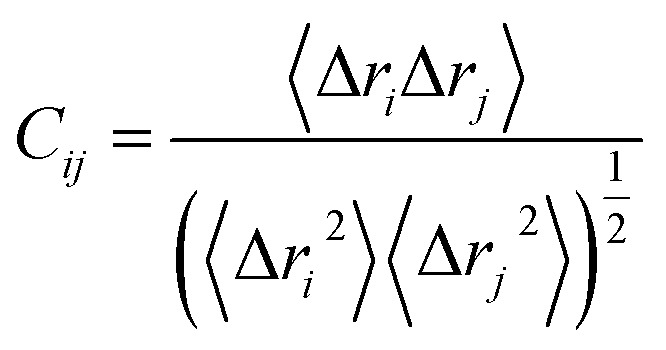
In this equation, Δ*r*_*i*_ represents the displacement of the ith C_α_ atom from its mean position. A *C*_*ij*_ value of +1 indicates strongly correlated movements between residues, whereas a value of −1 signifies highly anticorrelated motions within the trajectory. Deviation of the correlation value from +1 or −1 reflects motions that are neither fully correlated nor fully anticorrelated. The dynamic cross-correlation matrix (DCCM) was generated using the CPPTRAJ module within Amber 18. Subsequent plotting and analysis of the matrices were performed using Origin software (https://www.originlab.com).

#### 3.3.7. *In silico* drug-likeness predictions

The drug-likeness of a pharmacological agent can serve as a criterion to assess whether it possesses the necessary properties to function as an orally active herbicide. This assessment is based on the “Lipinski rule of five”, originally proposed by Lipinski and colleagues. *In silico* predictions of herbicide-likeness and toxicity for the investigated ligands were performed using DATA Warrior and the Swiss ADME predictor.^71^ The DATA Warrior program evaluates various parameters for each chemical, including solubility, cLog *P*, total polar surface area (TPSA), hydrophilicity (log *P*), molecular mass, as well as potential for mutagenicity, toxicity, irritancy, and reproductive effects. Meanwhile, the Swiss ADME predictor provides information on the synthetic accessibility of the compounds and the number of hydrogen bond donors, hydrogen bond acceptors, and rotatable bonds.

## 4. Conclusion

In conclusion, a new series of phenyl 1,2,3-triazole–2-pyridylpiperazine derivatives was successfully synthesized through a CuAAC click chemistry protocol. Numerous compounds exhibited encouraging antiproliferative activity against HCT-116, HepG-2, and MCF-7 cancer cell lines, with compound 15 demonstrating the most potent and consistent effects. Computational studies validated the stable binding of the lead compound within the EGFR active site and elucidated the key interactions that contribute to its activity. *In silico* pharmacokinetic and drug-likeness evaluations confirmed that the most potent compounds, 15 and 18, possess favorable drug-like properties and comply with Lipinski's rule of five, supporting their potential as orally bioavailable anticancer drug candidates. Compounds with Lipinski violations 19 and 23 were appropriately identified as non-drug-like and deprioritized. The proposed mechanism is based on computational predictions and requires further biochemical validation.

## Conflicts of interest

There are no conflicts to declare.

## Supplementary Material

RA-016-D6RA01413E-s001

## Data Availability

The data supporting this article have been included as part of the supplementary information (SI). Supplementary information is available. See DOI: https://doi.org/10.1039/d6ra01413e.
