# Supplementary material for: Click chemistry of phenyl 1,2,3-triazole–2-pyridylpiperazine hybrids: synthesis, targeted anticancer activity, molecular modeling and computational studies
Source: RSC Adv. 2026 May 14;16(28):25330–47. doi: 10.1039/d6ra01413e (PMC13182531; doi:10.1039/d6ra01413e)
Supplement: RA-016-D6RA01413E-s001 [file RA-016-D6RA01413E-s001.pdf]

Supplementary Information (SI) for RSC Advances.
This journal is © The Royal Society of Chemistry 2026

Click Chemistry of Phenyl 1,2,3-Triazole–2-Pyridylpiperazine Hybrids: Synthesis, Targeted Anticancer Activity, Molecular Modeling and Computational Studies

Tamer El Malah[a] * and Ahmed A. El-Rashedy[b,c]

[a] *Photochemistry Department, Chemical Industries Research Institute, National Research Centre, 33 El Buhouth Street, P.O. Box 12622, Cairo, Egypt*
[b] *Chemistry of Natural and Microbial Products Department, National Research Centre, Dokki, 12622 Cairo, Egypt*
[c] *Department of Organic and Medicinal Chemistry, Faculty of Pharmacy, University of Sadat City, Monofia 32897, Egypt*
*Corresponding author: tmara_nrc3000@yahoo.com (Tamer El Malah)

Table of Contents

Figure S1. ^{1}H NMR spectrum of compound **13** (400 MHz, $CDCl_3$, 25 °C)………… …... **3**

Figure S2. ^{13}C NMR spectrum of compound **13** (100 MHz, $CDCl_3$, 25 °C)………… ….. **3**

Figure S3. ^{1}H NMR spectrum of compound **14** (400 MHz, $CDCl_3$, 25 °C)………… …. **4**

Figure S4. ^{13}C NMR spectrum of compound **14** (100 MHz, $CDCl_3$, 25 °C)………… …… **4**

Figure S5. ^{1}H NMR spectrum of compound **15** (400 MHz, $CDCl_3$, 25 °C)………… ……... **5**

Figure S6. ^{13}C NMR spectrum of compound **15** (100 MHz, $CDCl_3$, 25 °C)………… …….. **5**

Figure S7. ^{1}H NMR spectrum of compound **16** (400 MHz, $CDCl_3$, 25 °C)……… …………... **6**

Figure S8. ^{13}C NMR spectrum of compound **16** (100 MHz, $CDCl_3$, 25 °C)………… …….. **6**

Figure S9. ^{1}H NMR spectrum of compound **17** (400 MHz, $CDCl_3$, 25 °C)……… ………... **7**

Figure S10. ^{13}C NMR spectrum of compound **17** (100 MHz, $CDCl_3$, 25 °C)…… ……… **7**

Figure S11. ^{1}H NMR spectrum of compound **18** (400 MHz, $CDCl_3$, 25 °C)……… ……. **8**

Figure S12. ^{13}C NMR spectrum of compound **18** (100 MHz, $CDCl_3$, 25 °C)…… ……… **8**

Figure S13. ^{1}H NMR spectrum of compound **19** (400 MHz, $CDCl_3$, 25 °C)……… ………... **9**

Figure S14. ^{13}C NMR spectrum of compound **19** (100 MHz, $CDCl_3$, 25 °C)……… …….. **9**

Figure S15. ^{1}H NMR spectrum of compound **20** (400 MHz, $CDCl_3$, 25 °C)………… ……... **10**

Figure S16. ^{13}C NMR spectrum of compound **20** (100 MHz, $CDCl_3$, 25 °C)……… …… **10**

Figure S17. ^{1}H NMR spectrum of compound **21** (400 MHz, $CDCl_3$, 25 °C)……… ………. **11**

Figure S18. ^{13}C NMR spectrum of compound **21** (100 MHz, $CDCl_3$, 25 °C)……… ……… **11**

Figure S19. ^{1}H NMR spectrum of compound **22** (400 MHz, $CDCl_3$, 25 °C)……… ……. **12**

Figure S20. ^{13}C NMR spectrum of compound **22** (100 MHz, $CDCl_3$, 25 °C)……… ……… **12**

Figure S21. ^{1}H NMR spectrum of compound **23** (400 MHz, $CDCl_3$, 25 °C)……… ………. **13**

Figure S22. ^{13}C NMR spectrum of compound **23** (100 MHz, $CDCl_3$, 25 °C)……… ……… **13**

Figuer 23s. Atomic charge calculation for compounds **13-23.** **14-16**

Figure 24s:. The Concept and Applications of POM Theory in the identification and optimization of pharmacophore sites of various classes of drugs, was developed by Prof. T. Ben Hadda (Principal Inventor of POM Theory) in collaboration with NCI and TAACF of theUSA. **17**

Compound 13

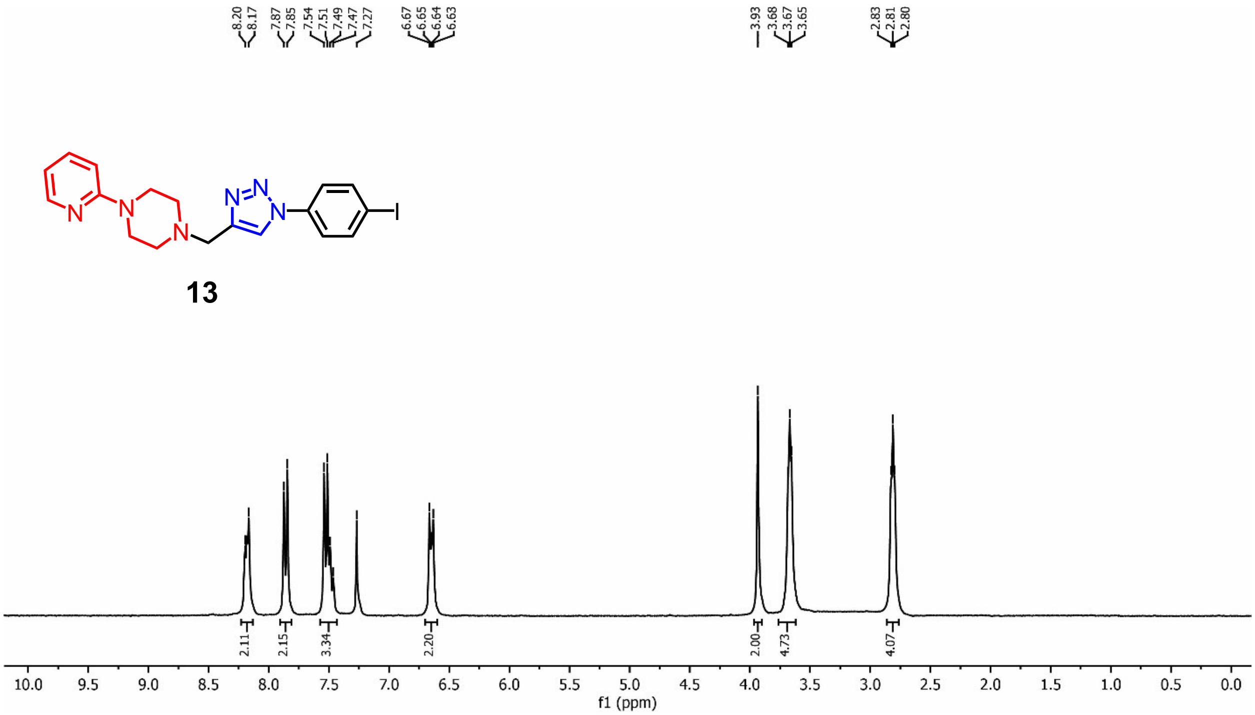

Figure S1. ^{1}H NMR spectrum of compound **13** (400 MHz, $CDCl_3$, 25 °C)

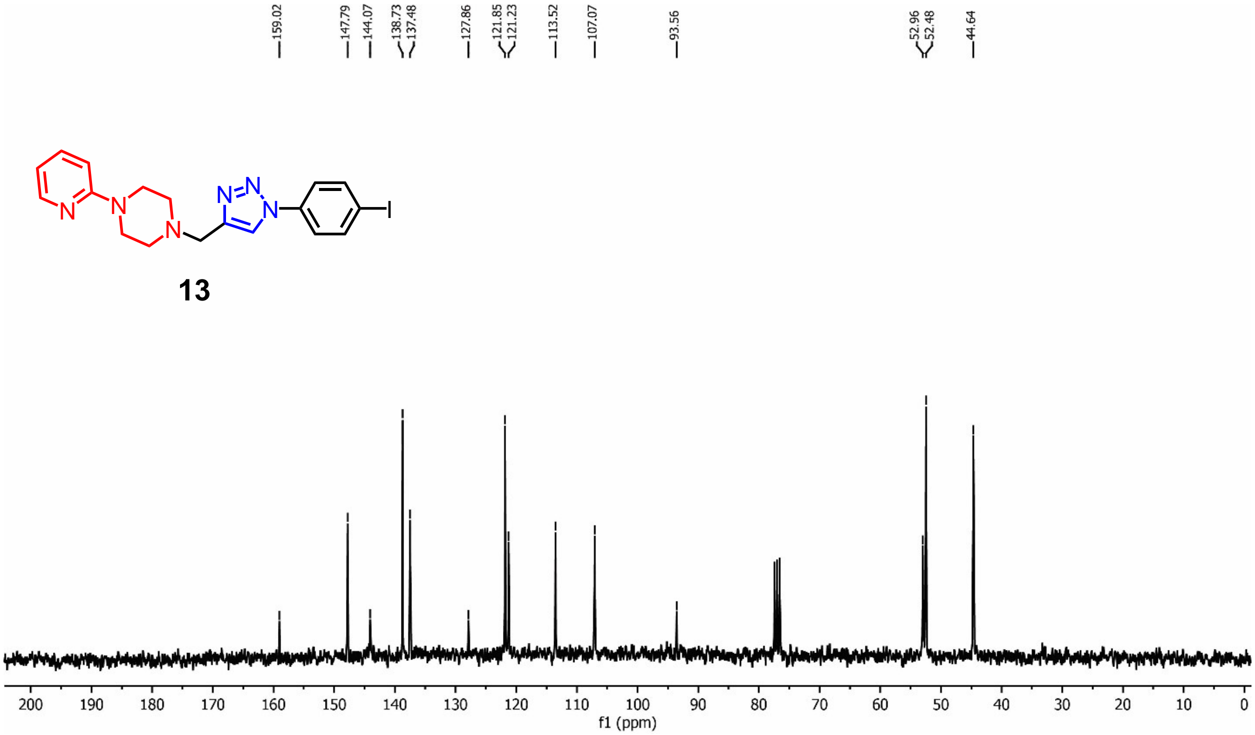

Figure S2. ^{13}C NMR spectrum of compound **13** (100 MHz, $CDCl_3$, 25 °C)

Compound 14

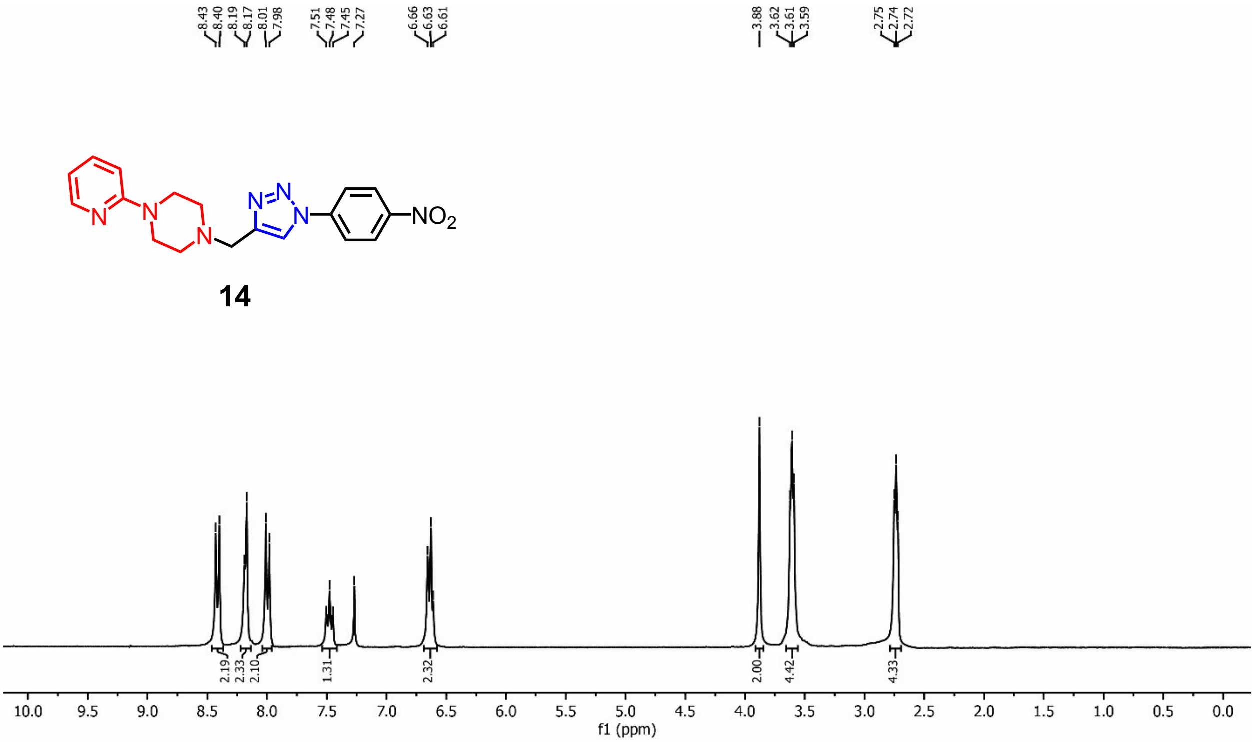

Figure S3. ^{1}H NMR spectrum of compound **14** (400 MHz, $CDCl_3$, 25 °C)

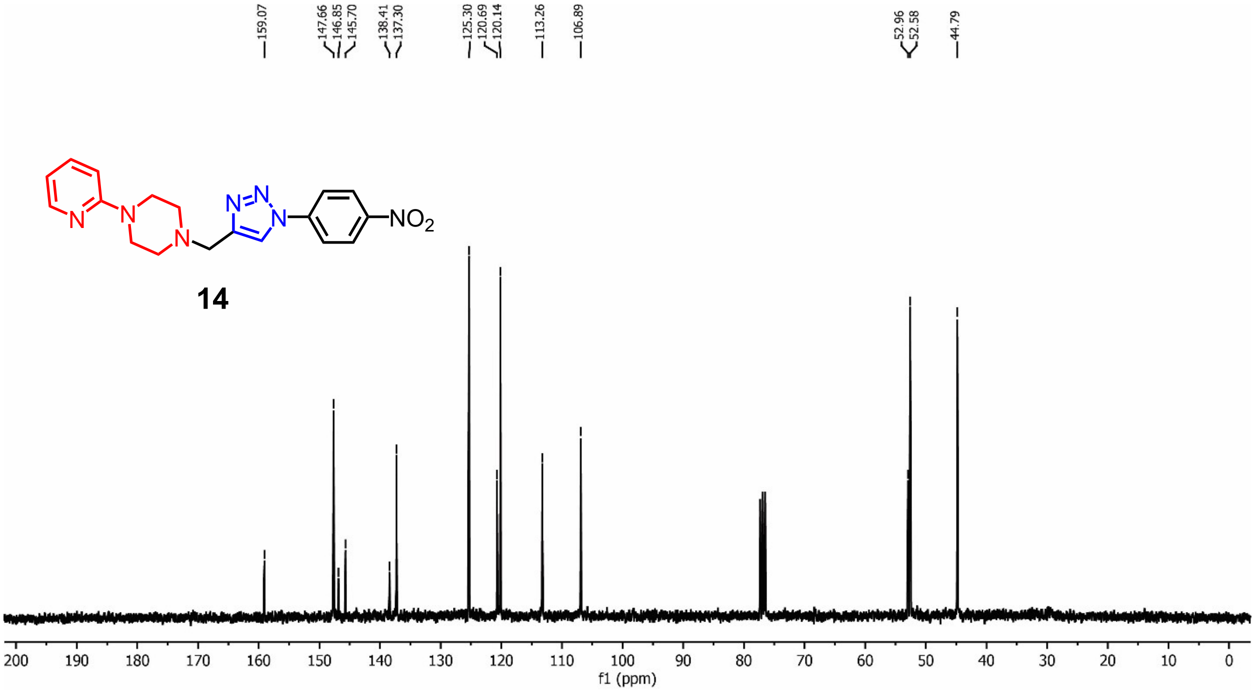

Figure S4. ^{13}C NMR spectrum of compound **14** (100 MHz, $CDCl_3$, 25 °C)

Compound 15

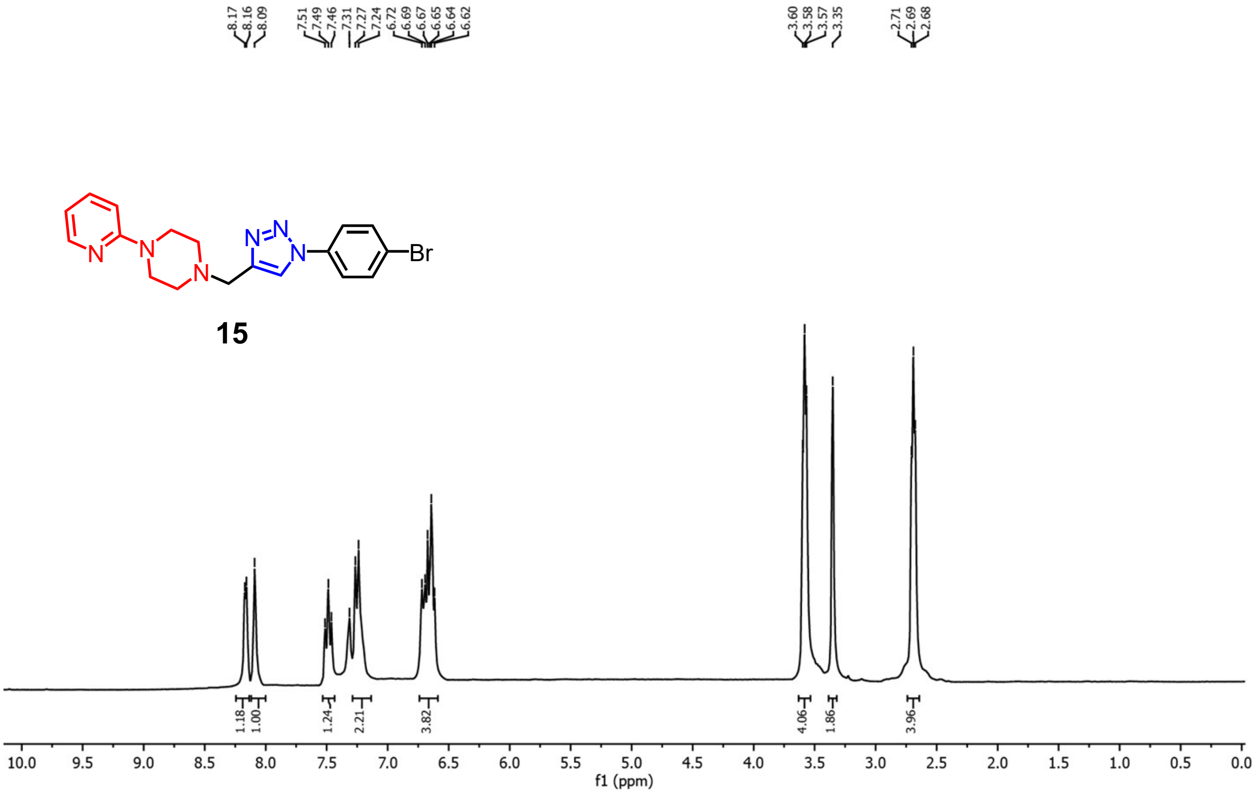

Figure S5. ^{1}H NMR spectrum of compound **15** (400 MHz, $CDCl_3$, 25 °C)

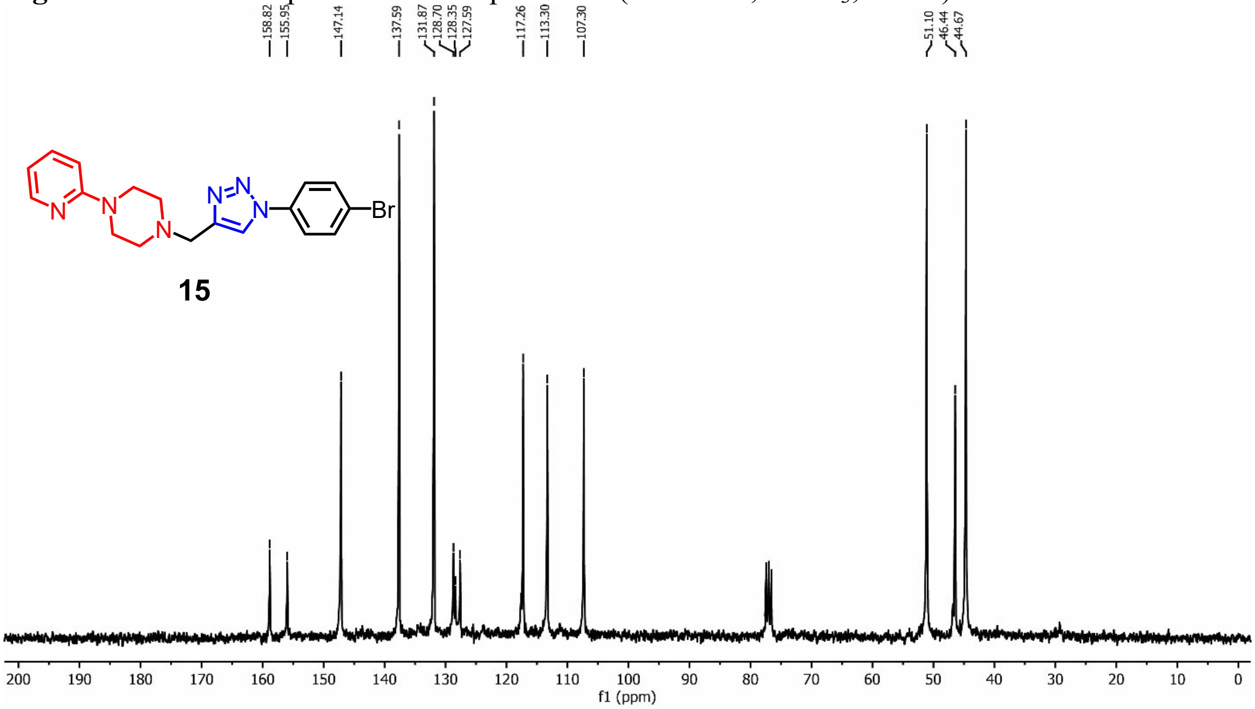

Figure S6. ^{13}C NMR spectrum of compound **15** (100 MHz, $CDCl_3$, 25 °C)

Compound 16

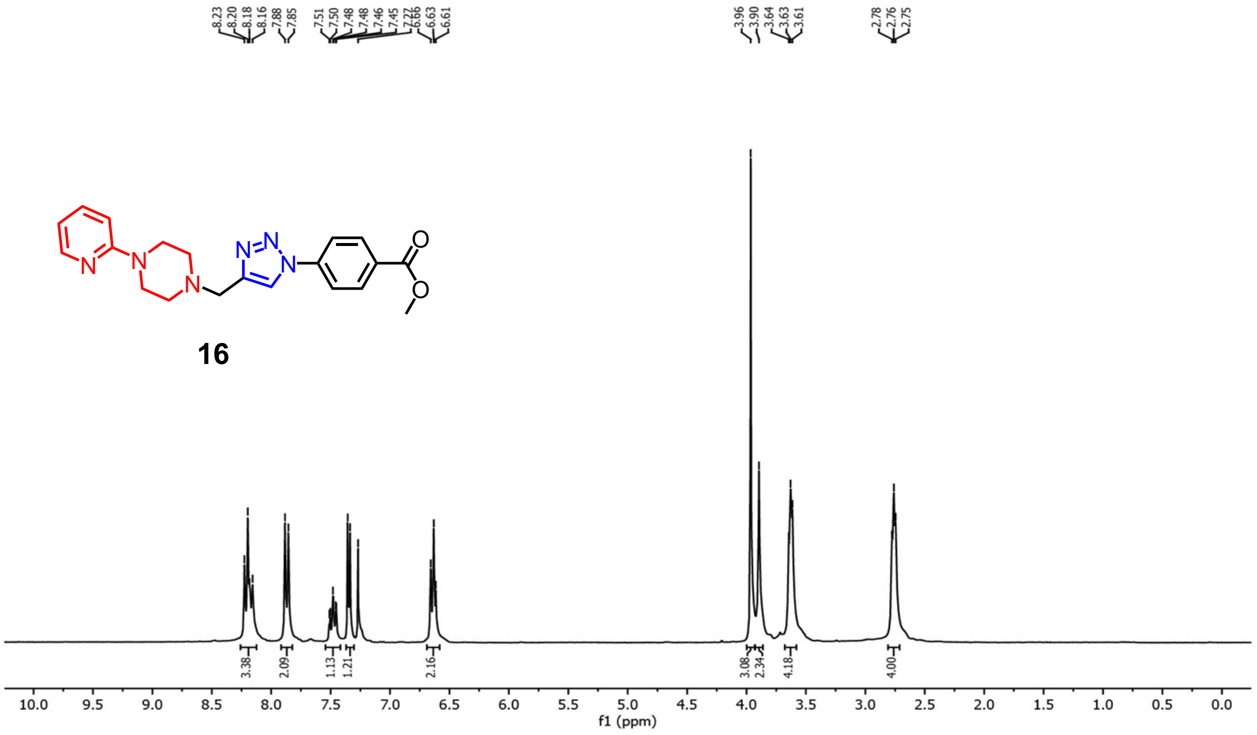

Figure S7. ^{1}H NMR spectrum of compound **16** (400 MHz, $CDCl_3$, 25 °C)

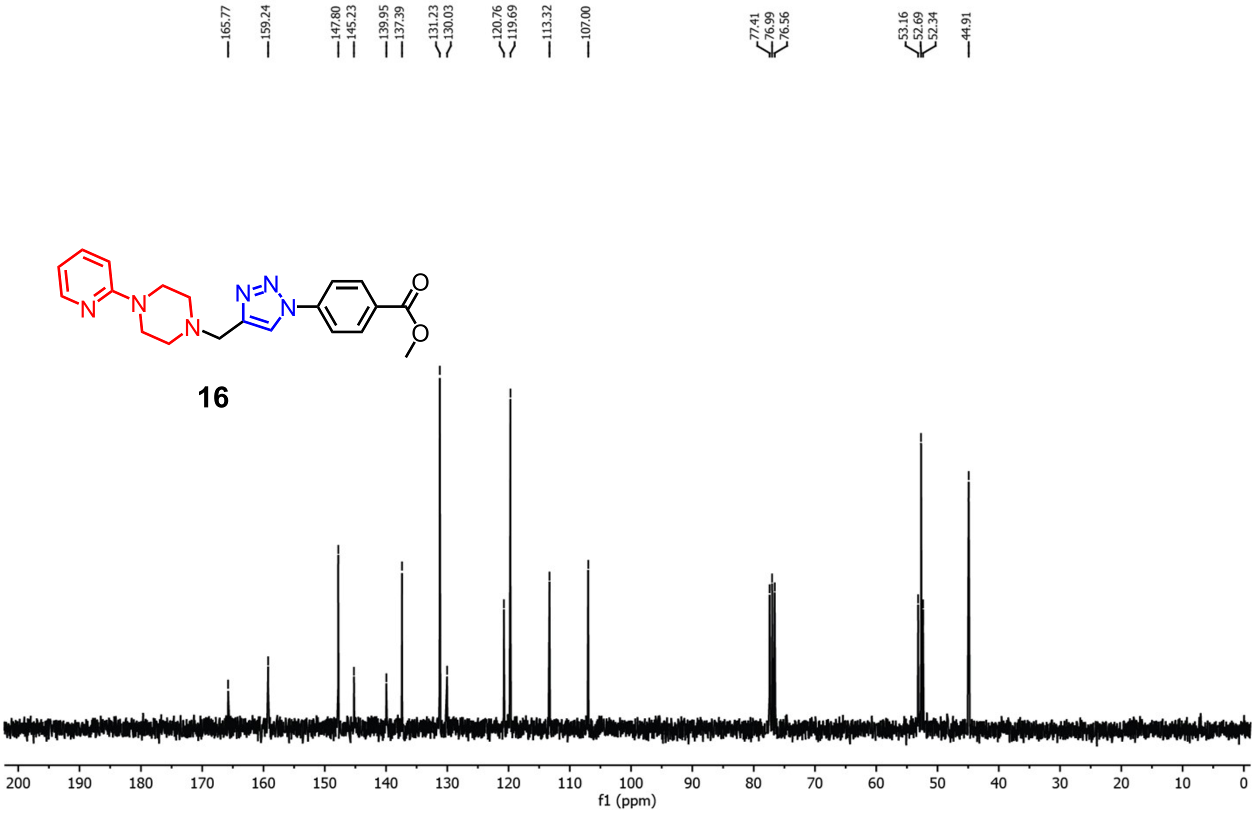

Figure S8. ^{13}C NMR spectrum of compound **16** (100 MHz, $CDCl_3$, 25 °C)

Compound 17

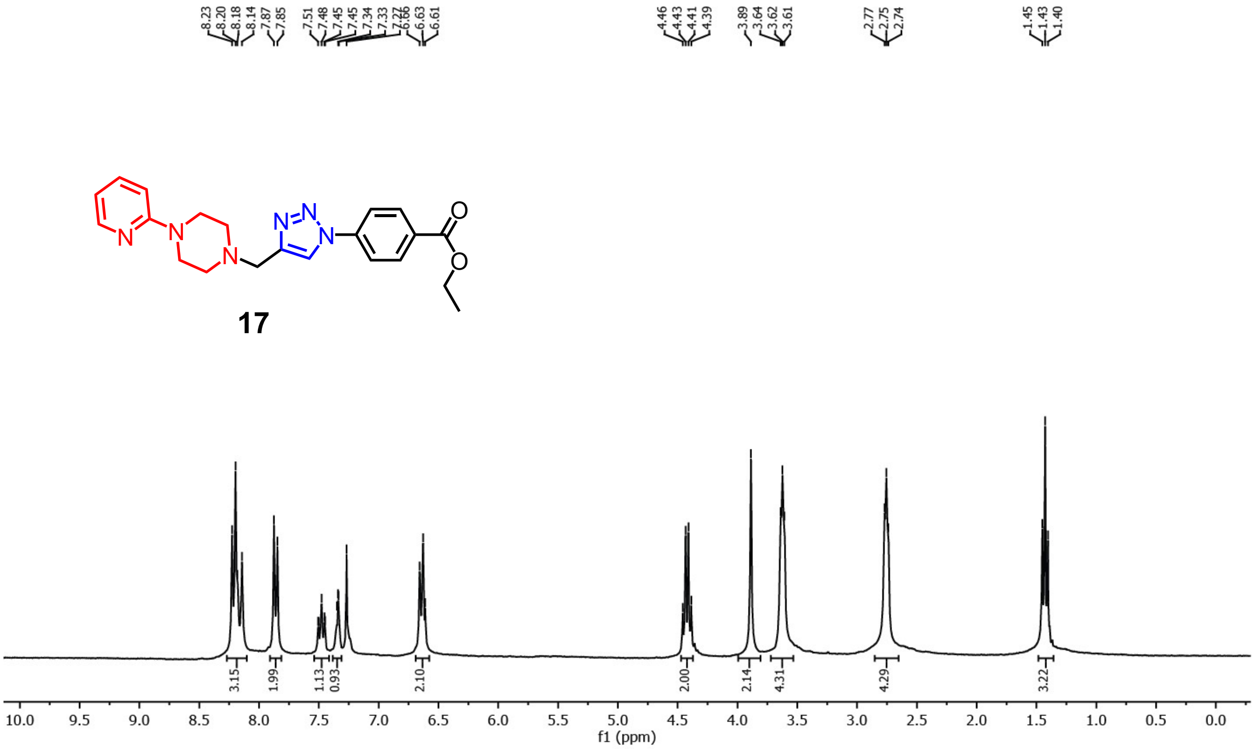

Figure S9. ^{1}H NMR spectrum of compound **17** (400 MHz, $CDCl_3$, 25 °C)

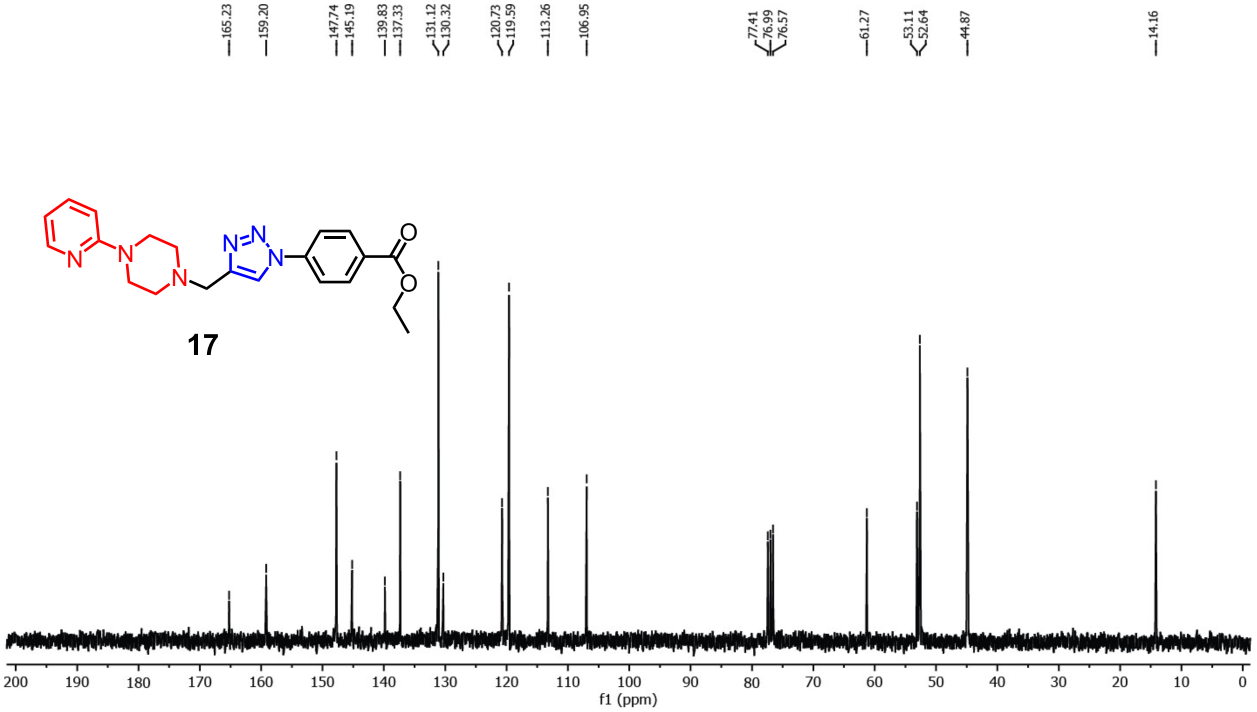

Figure S10. ^{13}C NMR spectrum of compound **17** (100 MHz, $CDCl_3$, 25 °C)

Compound 18

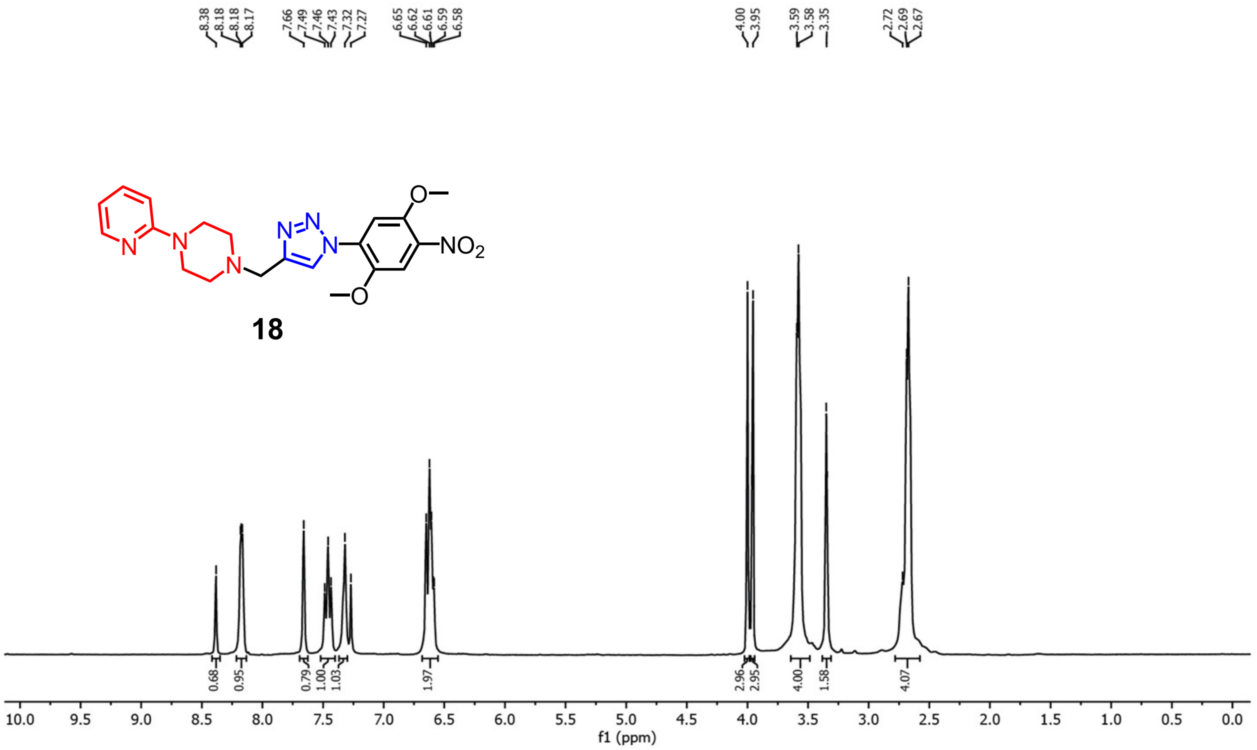

Figure S11. ^{1}H NMR spectrum of compound **18** (400 MHz, $CDCl_3$, 25 °C)

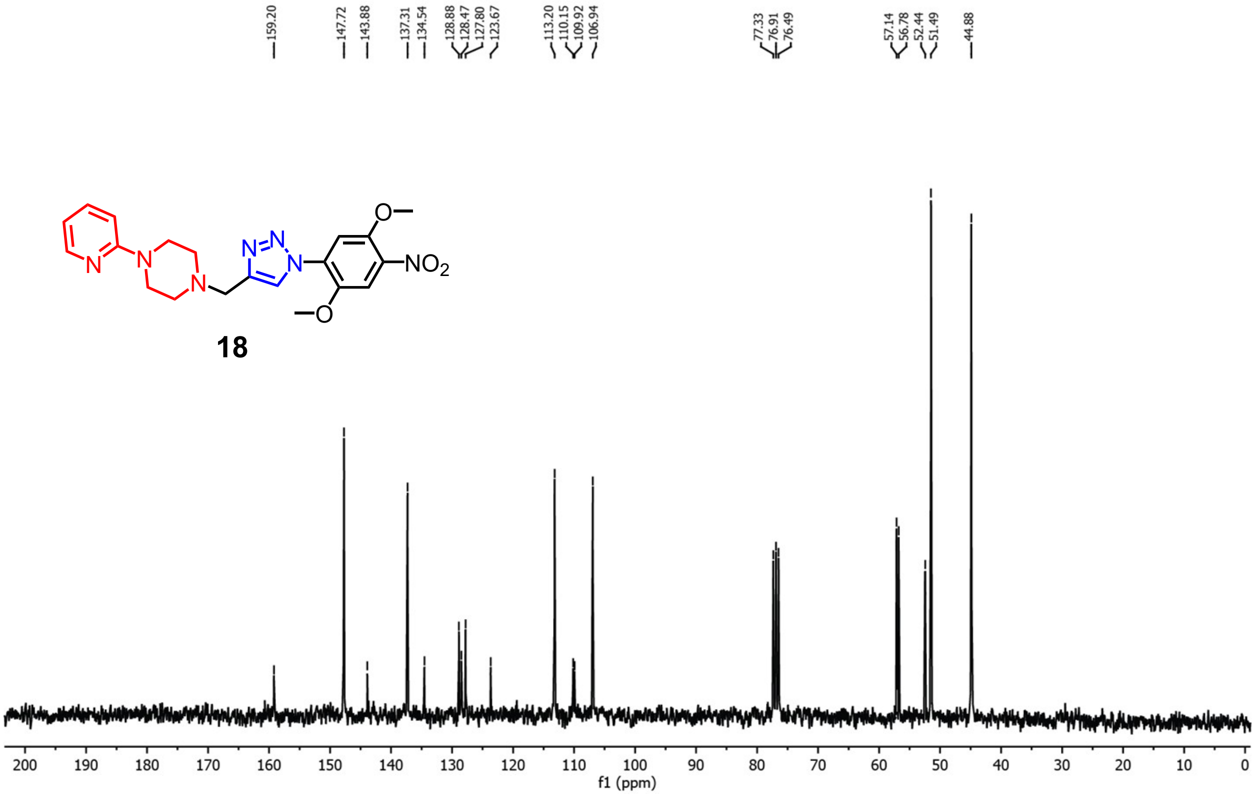

Figure S12. ^{13}C NMR spectrum of compound **18** (100 MHz, $CDCl_3$, 25 °C)

Compound 19

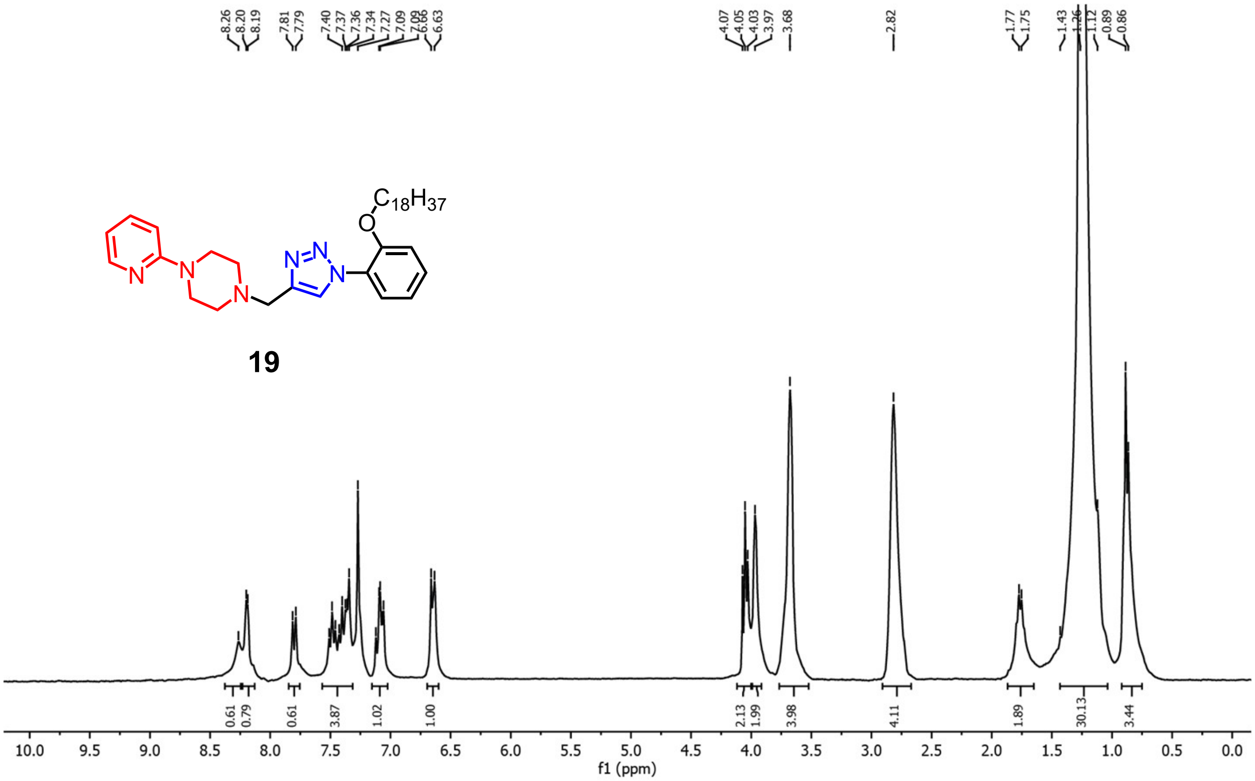

Figure S13. ^{1}H NMR spectrum of compound **19** (400 MHz, $CDCl_3$, 25 °C)

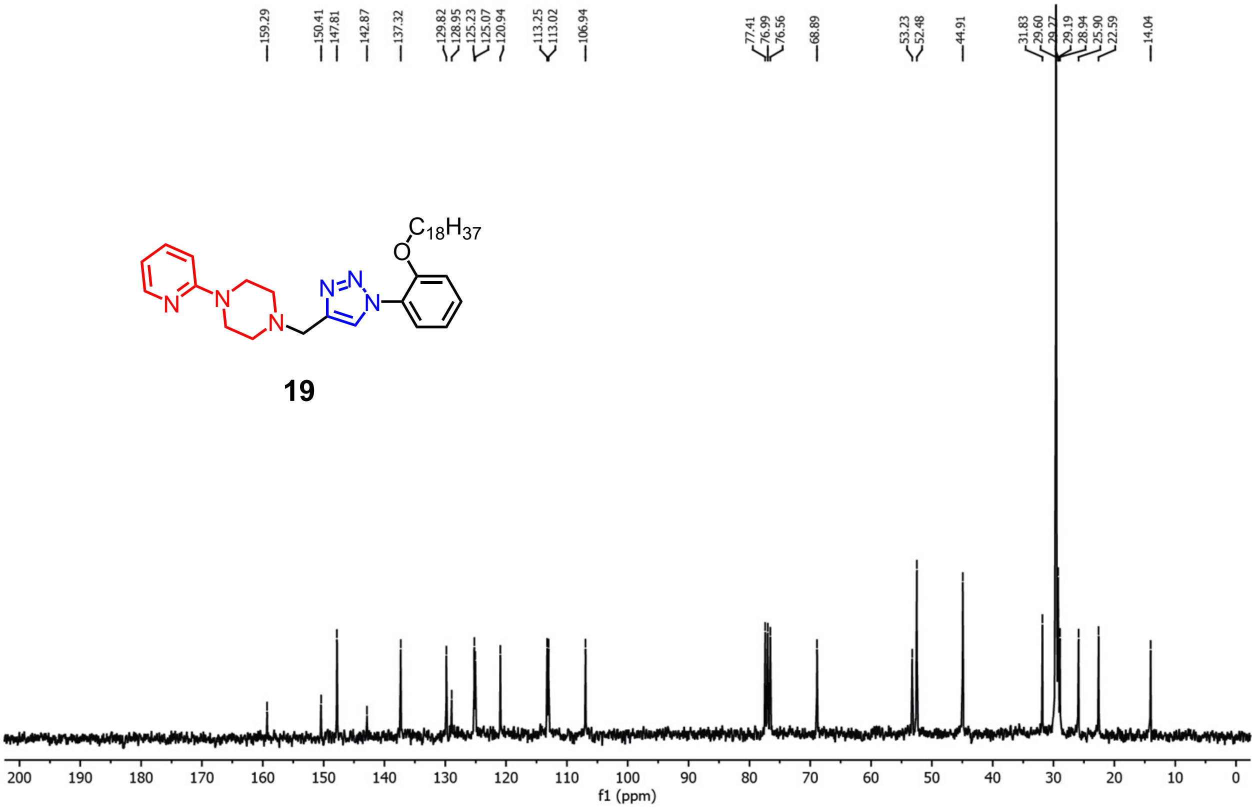

Figure S14. ^{13}C NMR spectrum of compound **19** (100 MHz, $CDCl_3$, 25 °C)

Compound 20

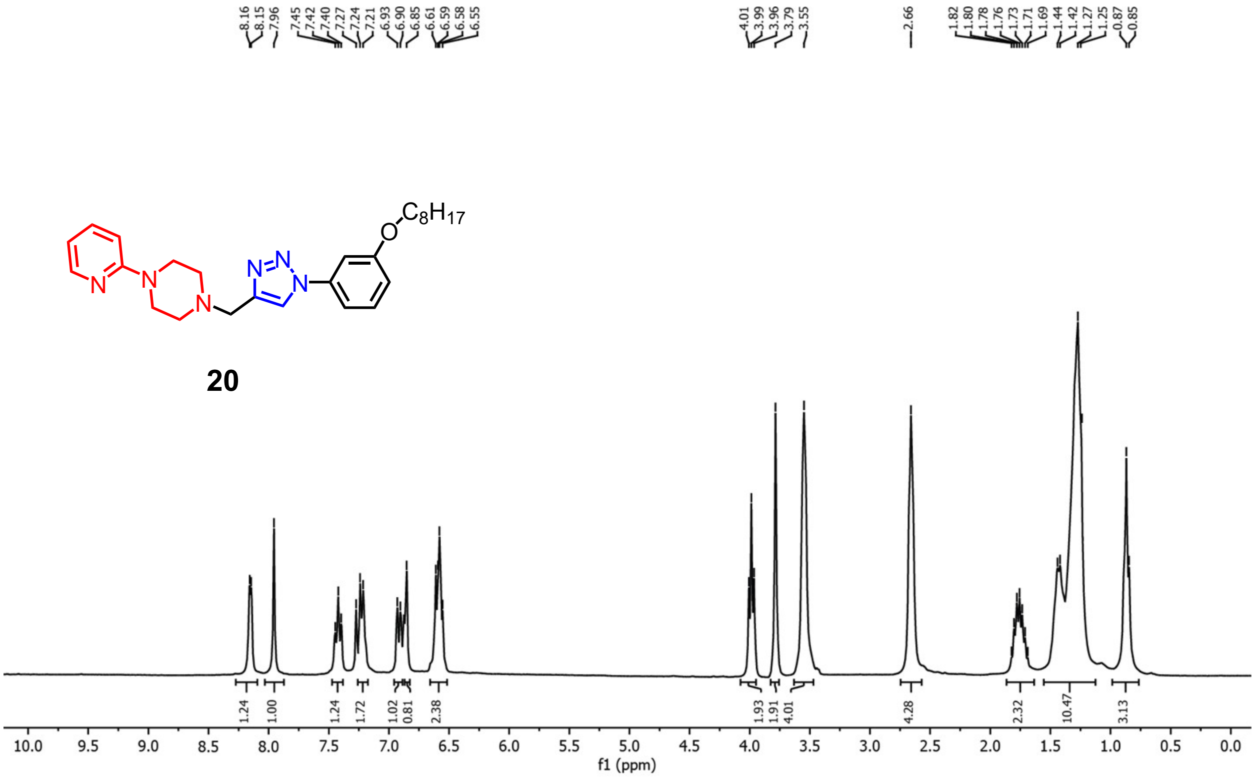

Figure S15. ^{1}H NMR spectrum of compound **20** (400 MHz, $CDCl_3$, 25 °C)

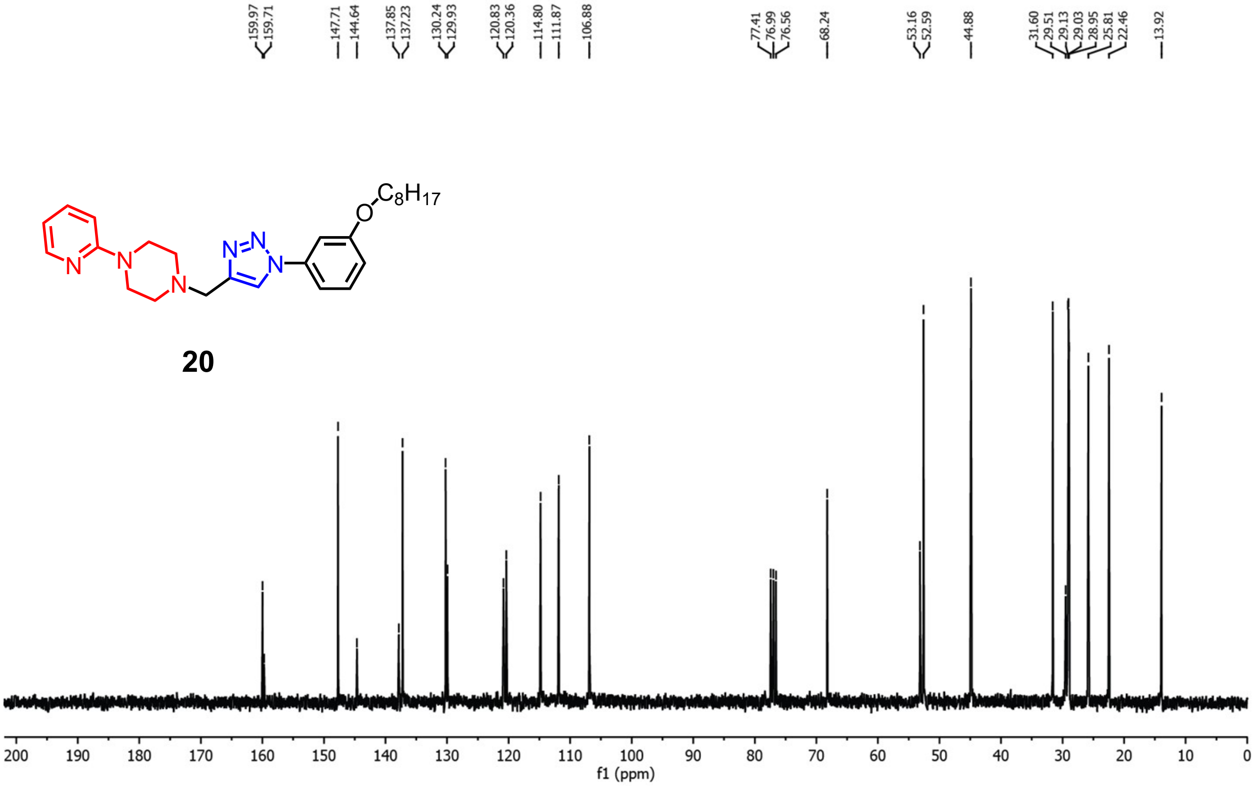

Figure S16. ^{13}C NMR spectrum of compound **20** (100 MHz, $CDCl_3$, 25 °C)

Compound 21

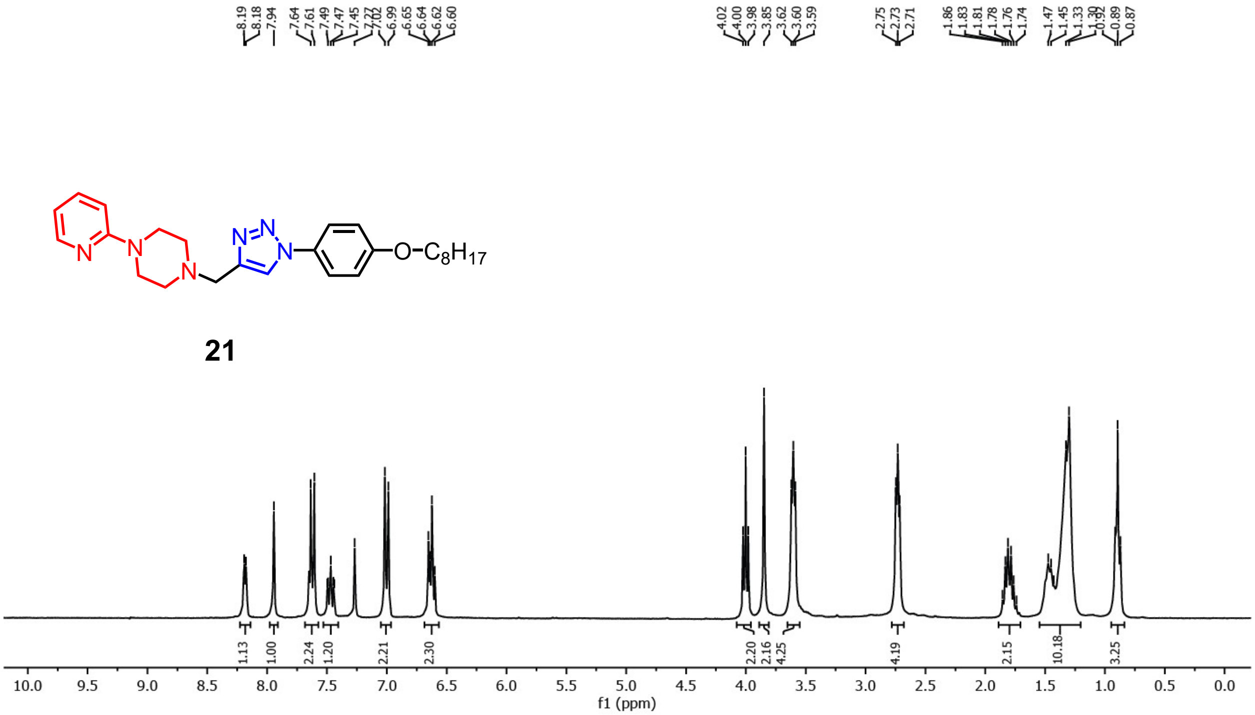

Figure S17. ^{1}H NMR spectrum of compound **21** (400 MHz, $CDCl_3$, 25 °C)

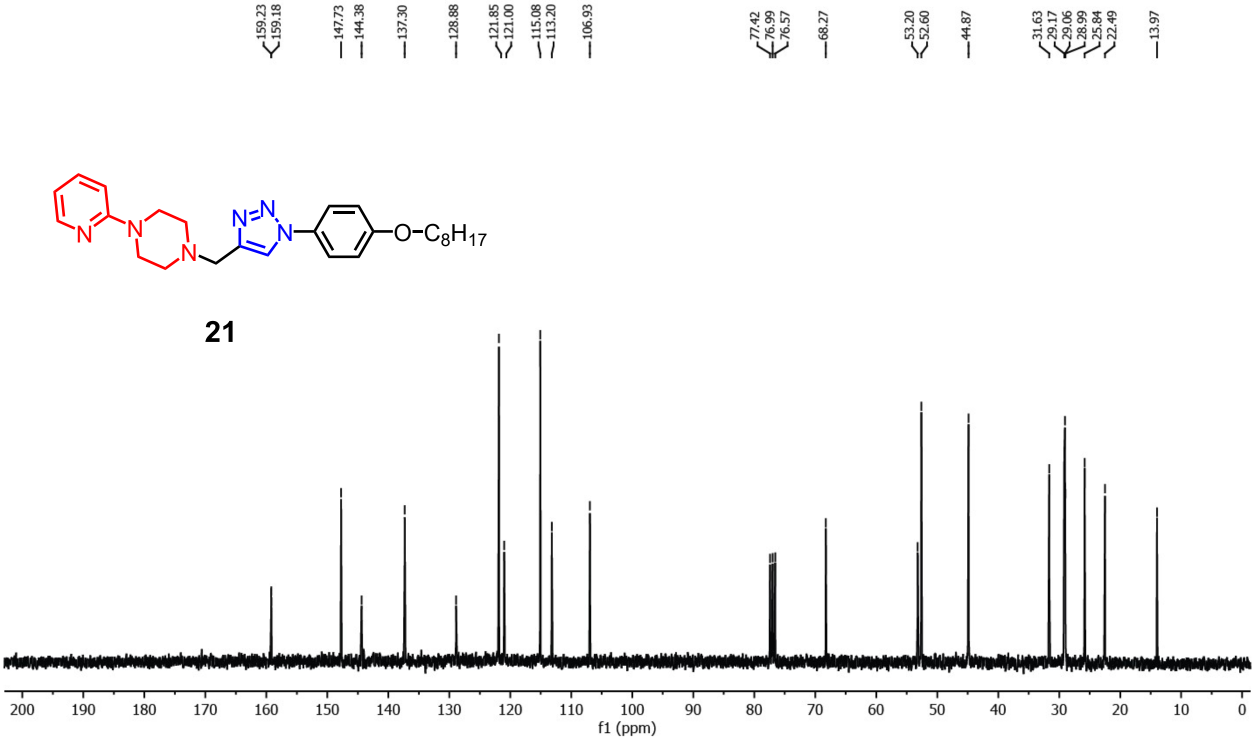

Figure S18. ^{13}C NMR spectrum of compound **21** (100 MHz, $CDCl_3$, 25 °C)

Compound 22

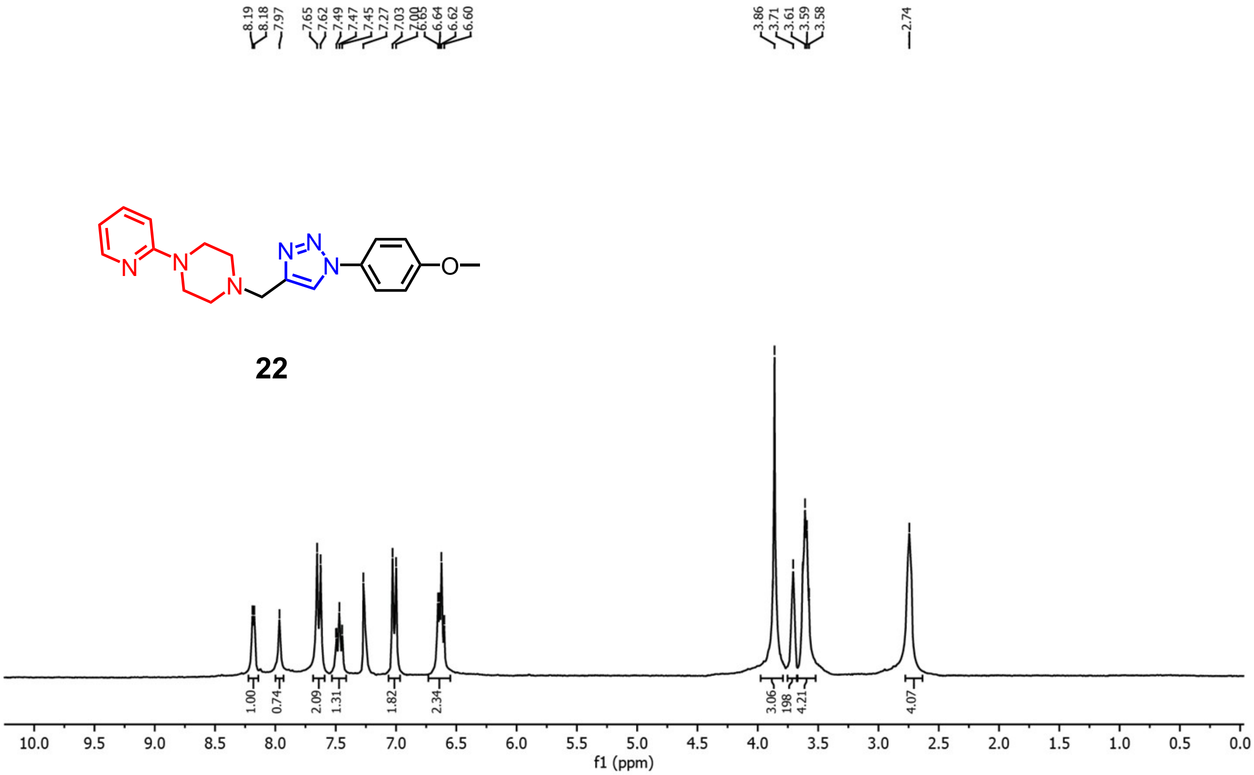

Figure S19. ^{1}H NMR spectrum of compound **22** (400 MHz, $CDCl_3$, 25 °C)

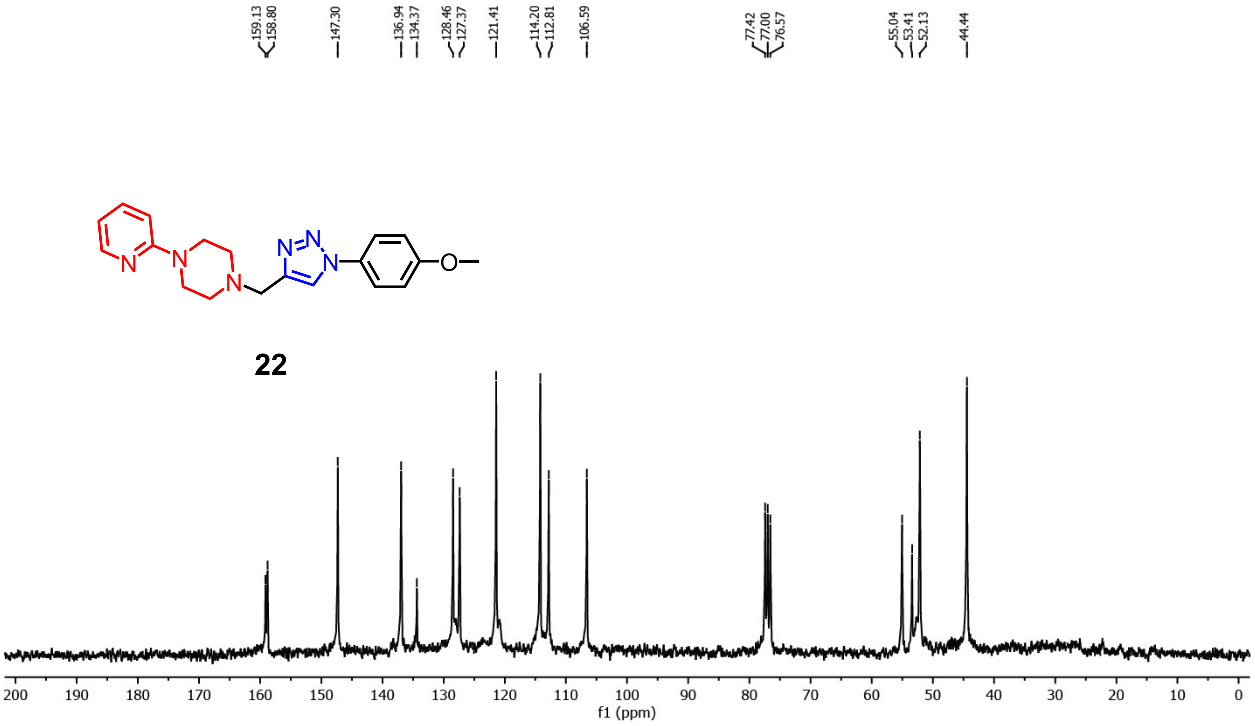

Figure S20. ^{13}C NMR spectrum of compound **22** (100 MHz, $CDCl_3$, 25 °C)

Compound 23

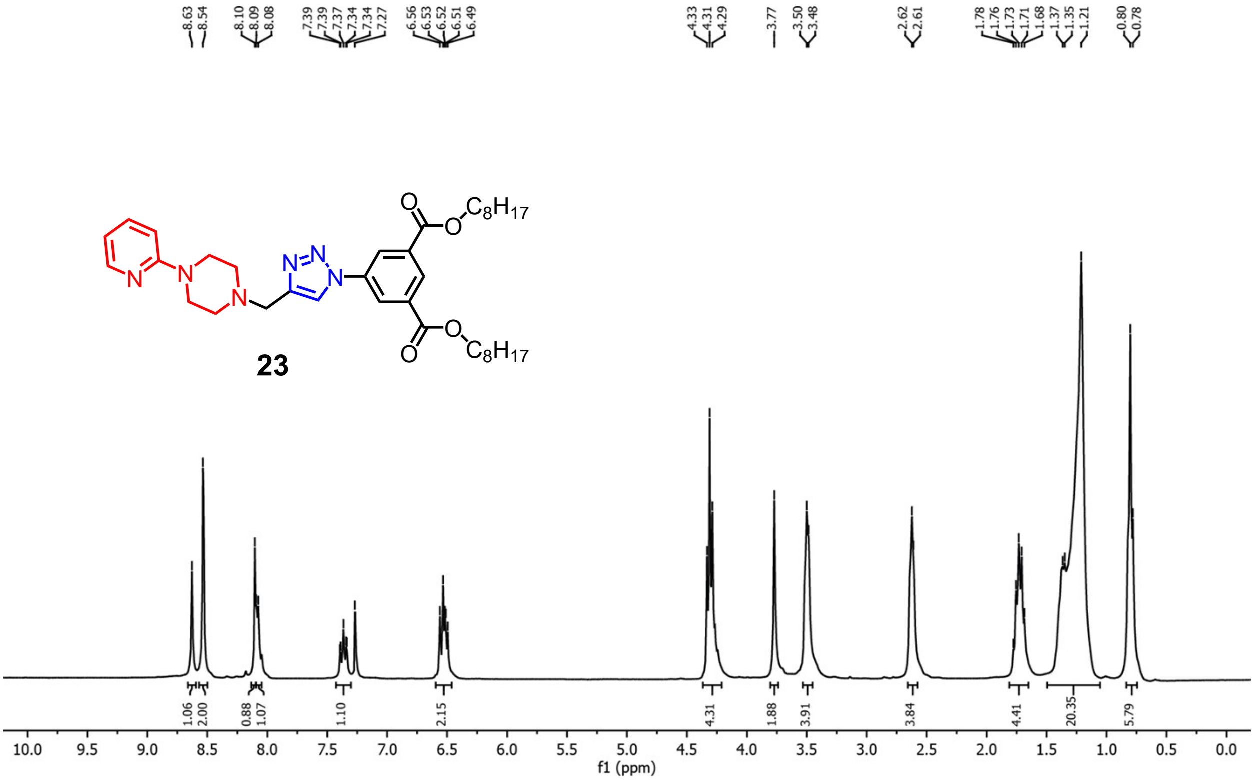

Figure S21. ^{1}H NMR spectrum of compound **23** (400 MHz, $CDCl_3$, 25 °C)

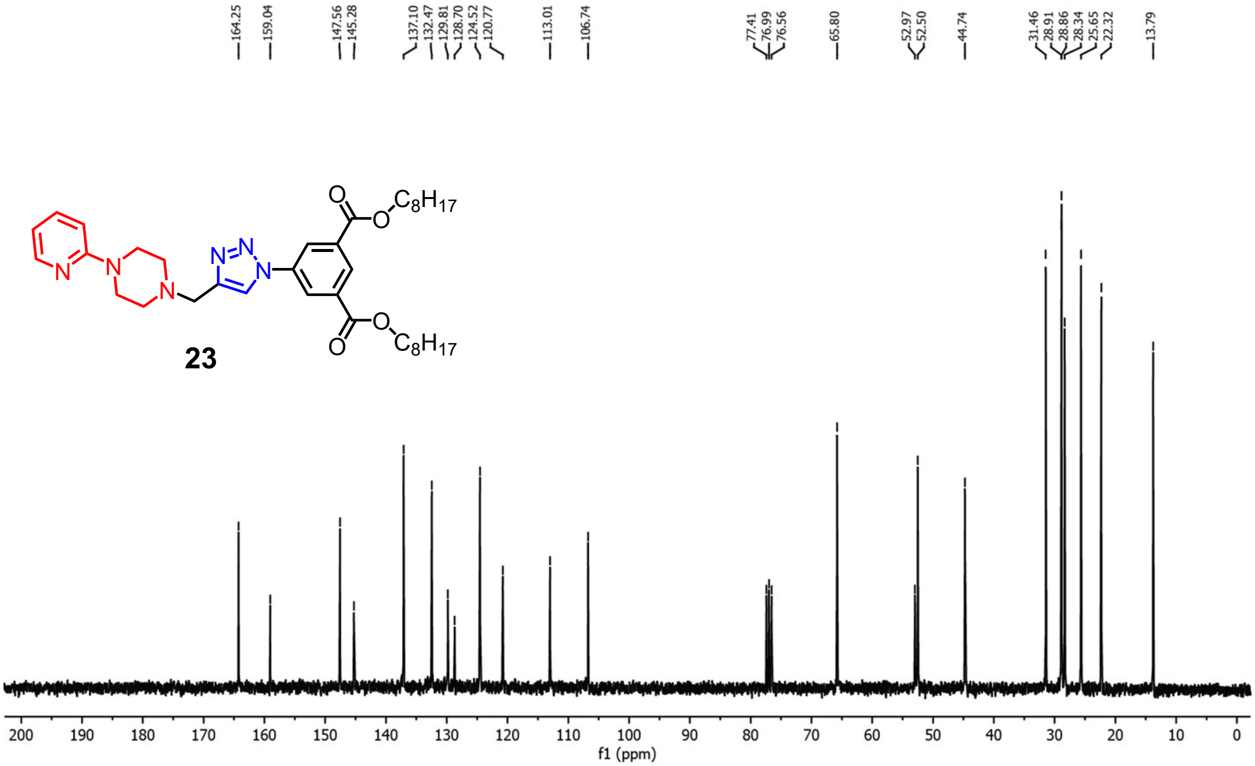

Figure S22. ^{13}C NMR spectrum of compound **23** (100 MHz, $CDCl_3$, 25 °

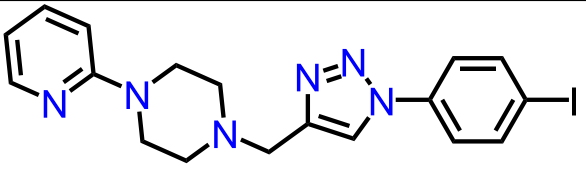

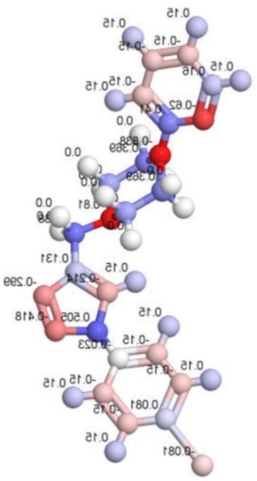

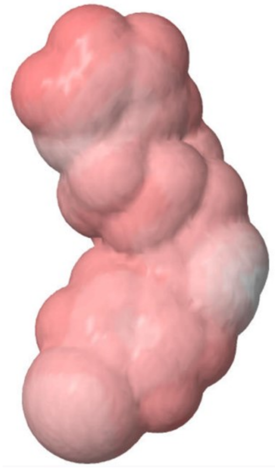

Compound 13

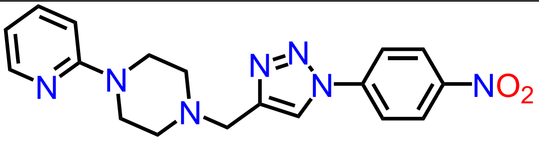

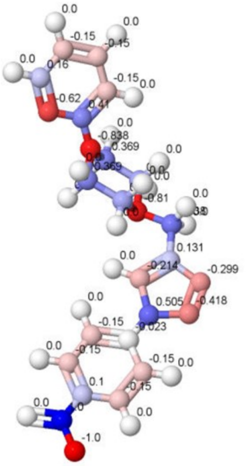

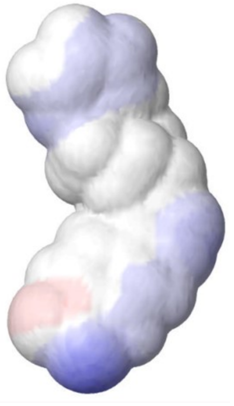

Compound 14

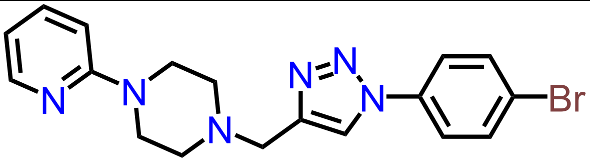

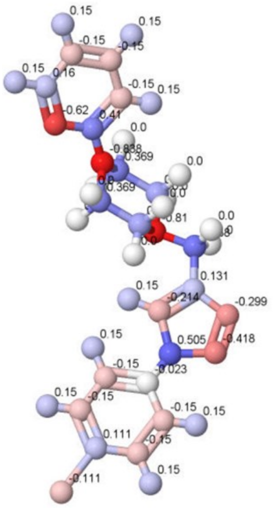

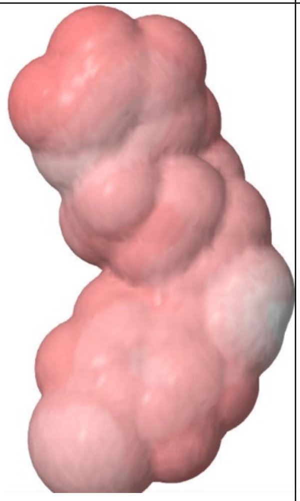

Compound 15

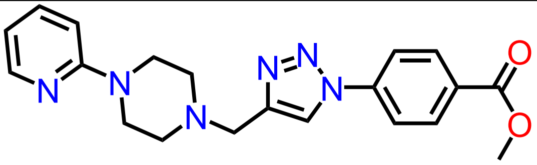

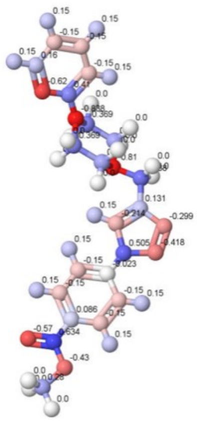

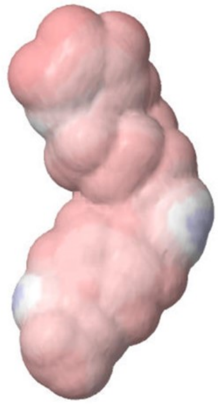

Compound 16

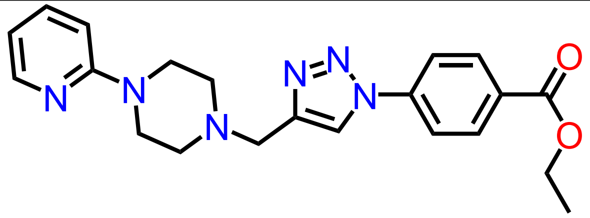	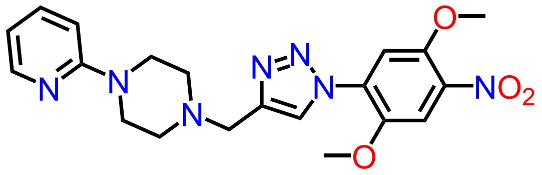
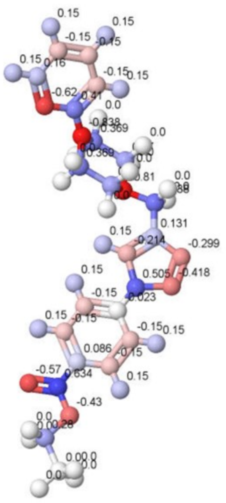 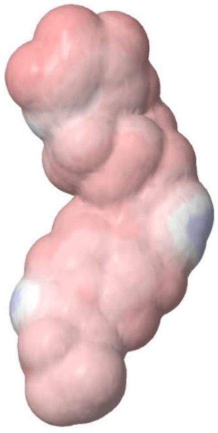	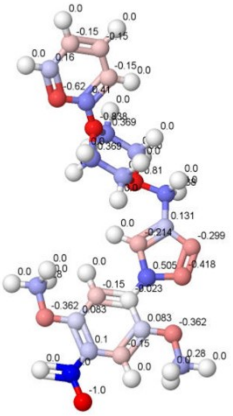 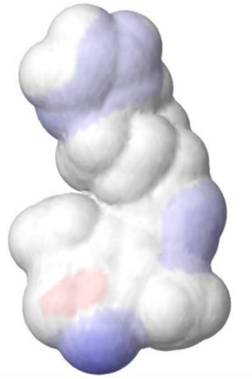
**Compound 17**	**Compound 18**
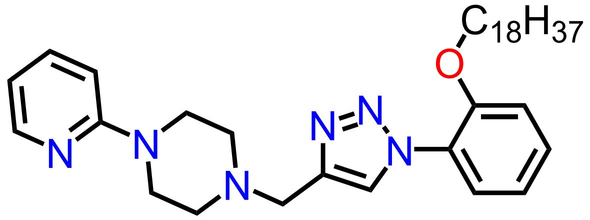	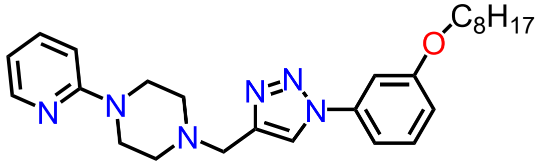
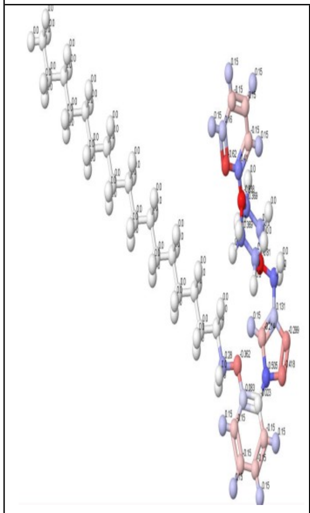 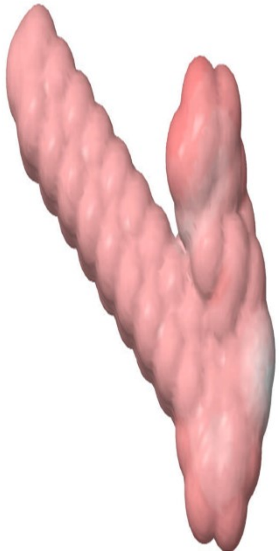	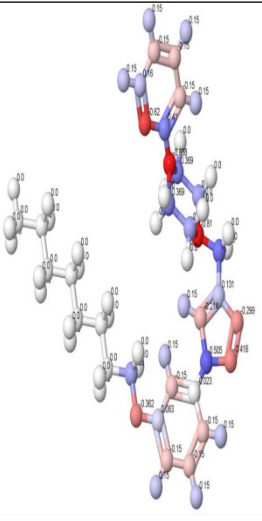 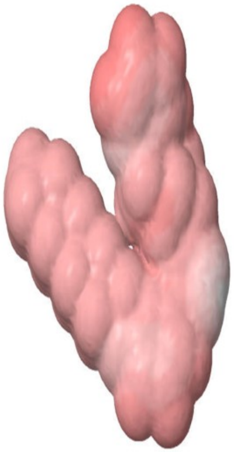

Compound 19		Compound 20	
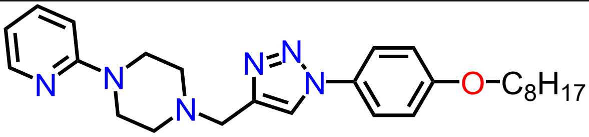		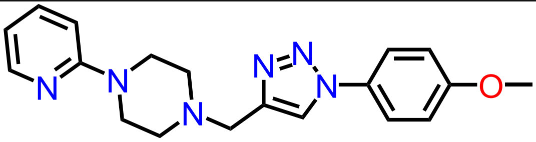	
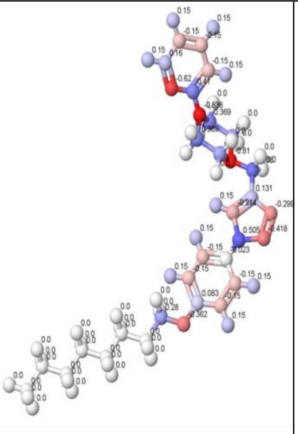	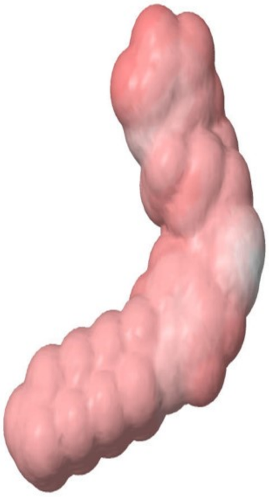	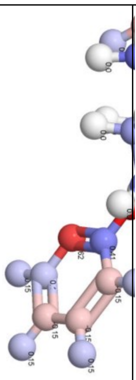	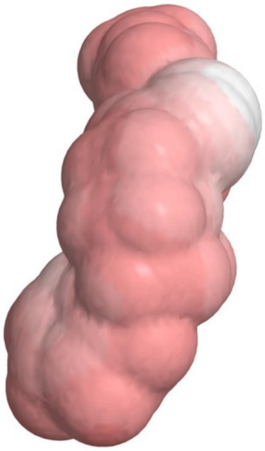
Compound 21		Compound 22	
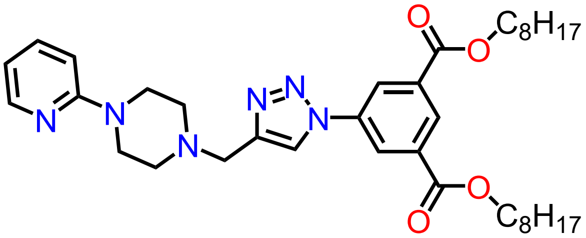			
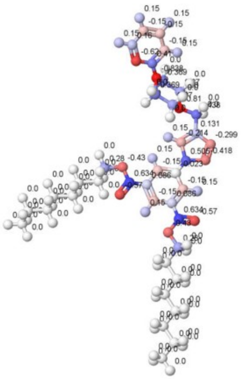		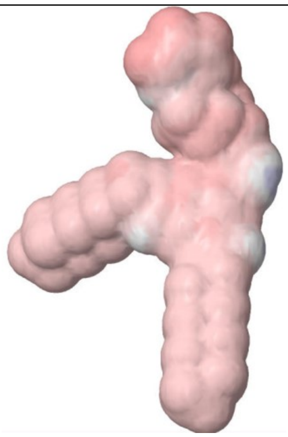	
Compound 23			

Figuer 23s : Atomic charge calculation for compounds **13-23**.

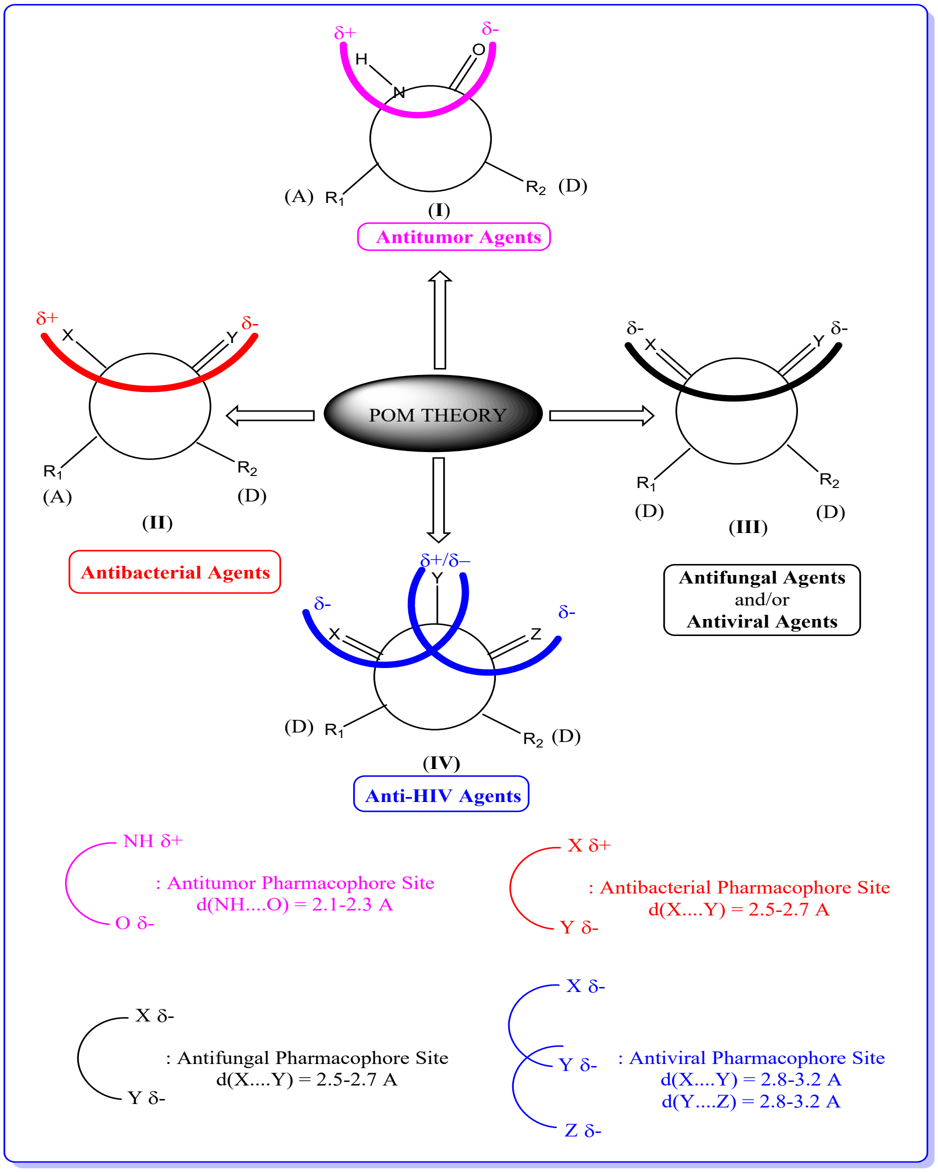

Figure 24s:.The Concept and Applications of POM Theory in the identification and optimization of pharmacophore sites of various classes of drugs, was developed by Prof. T. Ben Hadda (Principal Inventor of POM Theory) in collaboration with NCI and TAACF of theUSA.
